# The induction and function of the anti-inflammatory fate of T_H_17 cells

**DOI:** 10.1038/s41467-020-17097-5

**Published:** 2020-07-03

**Authors:** Hao Xu, Theodora Agalioti, Jun Zhao, Babett Steglich, Ramez Wahib, Maria Carolina Amezcua Vesely, Piotr Bielecki, Will Bailis, Ruaidhri Jackson, Daniel Perez, Jakob Izbicki, Paula Licona-Limón, Vesa Kaartinen, Jens Geginat, Enric Esplugues, Eva Tolosa, Samuel Huber, Richard A. Flavell, Nicola Gagliani

**Affiliations:** 1 Department of Immunobiology, School of Medicine, Yale University, New Haven, CT 06520 USA; 2 Department of General, Visceral and Thoracic Surgery, University Medical Center Hamburg-Eppendorf, 20246 Hamburg, Germany; 3 I. Department of Medicine, University Medical Center Hamburg-Eppendorf, 20246 Hamburg, Germany; 4 Department of Pathology and Laboratory Medicine, Perelman School of Medicine, University of Pennsylvania, Philadelphia, PA 19104 USA; 5 Division of Protective Immunity, The Children’s Hospital of Philadelphia, Philadelphia, PA 19104 USA; 6 Departamento de Biología Celular y del Desarrollo, Instituto de Fisiología Celular, Universidad Nacional Autónoma de México, D.F, México; 7 Biologic and Material Sciences, University of Michigan, 1011N. University Ave, Ann Arbor, MI 48109 USA; 8 INGM-National Institute of Molecular Genetics “Romeo ed Enrica Invernizzi”, Milan, Italy; 9 Department of Clinical Sciences and Community Health, Università degli studi di Milano, Milan, Italy; 10 Laboratory of Molecular and Cellular Immunology, Principe Felipe Research Center (CIPF), 46012 Valencia, Spain; 11 Institute of Immunology, University Medical Center Hamburg-Eppendorf, 20246 Hamburg, Germany; 12 Howard Hughes Medical Institute, Yale University School of Medicine, New Haven, CT USA; 13 Immunology and Allergy Unit, Department of Medicine, Solna, Karolinska Institute and University Hospital, Stockholm, Sweden

**Keywords:** Immunology, Transforming growth factor beta, Inflammation, Regulatory T cells, T-helper 17 cells

## Abstract

T_H_17 cells exemplify environmental immune adaptation: they can acquire both a pathogenic and an anti-inflammatory fate. However, it is not known whether the anti-inflammatory fate is merely a vestigial trait, or whether it serves to preserve the integrity of the host tissues. Here we show that the capacity of T_H_17 cells to acquire an anti-inflammatory fate is necessary to sustain immunological tolerance, yet it impairs immune protection against *S*. *aureus*. Additionally, we find that TGF-β signalling via Smad3/Smad4 is sufficient for the expression of the anti-inflammatory cytokine, IL-10, in T_H_17 cells. Our data thus indicate a key function of T_H_17 cell plasticity in maintaining immune homeostasis, and dissect the molecular mechanisms explaining the functional flexibility of T_H_17 cells with regard to environmental changes.

## Abstract (web summary)

CD4^+^ T helper cells producing IL-17A (T_H_17 cells) can take on pathogenic or anti-inflammatory functions in context-specific manners. Here the authors show that the anti-inflammatory fate of T_H_17 cells contributes, via TGF-β signaling and induction of IL-10, to host immune tolerance, but also simultaneously dampens protective immunity against *S. aureus*.

## Introduction

The consequences of dysfunctional immune adaptation are clear: the immune cells either fail to mount a protective response to lethal infections, or they overreact to non-harmful antigens and in doing so, induce disparate immune mediated inflammatory diseases (IMIDs). It is therefore both fundamentally and clinically relevant to reveal the molecular mechanisms underlying this phenomenon of immune adaptation.

T_H_17 cells—a subset of CD4^+^ T cells—exemplify immune adaptation. On the one hand, T_H_17 cells can have a full effector function protecting the host from pathogens with the associated risk of developing IMIDs^1–5^. On the other hand, T_H_17 cells can also acquire an anti-inflammatory fate characterized by secretion of the anti-inflammatory cytokine, IL-10^6–8^. However, the relevance of this T_H_17 anti-inflammatory fate and the consequences of its impairment have not yet been fully explored. T_H_17 cells are abundant in the intestine^6^, but it remains to be tested whether by acquiring an anti-inflammatory fate, they contribute to intestinal immune homeostasis by blocking the development of a pathological response and preserving the tissue integrity. Furthermore, whether this anti-inflammatory fate negatively impacts T_H_17 mediated immunity to pathogens is also unknown.

Finally, the exact molecular mechanism behind this cellular adaptation also remains to be clarified. Even though the key molecular pathways for the differentiation of CD4^+^ T cells into T_H_17 cells have been studied extensively^9–12^, investigation into the mechanisms that promote the anti-inflammatory fate of mature T_H_17 cells has just begun^7,13–16^. On the basis of in vitro screenings, we proposed that TGF-β controls the expression of IL-10 in CD4^+^ T cells during their in vitro differentiation into T_H_17 cells^7,17^. However, whether TGF-β also plays a key role in vivo and on already matured T_H_17 cells, and through which molecular signalling, remain to be studied.

It is known that upon engagement of TGF-β with TGFBR1/R2 on CD4^+^ T cells, TGFBR2 trans phosphorylates TGFBR1, which propagates signalling by phosphorylating Smad2 and Smad3. Once Smad2 and Smad3 form a heteromeric complex with the common mediator Smad4, they translocate to the nucleus to trans-activate several genes. It has been shown that while Smad2 promotes RORγt activity (i.e. the key transcriptional factor of T_H_17 cell differentiation), Smad3 impairs it^18,19^. Furthermore, Smad4 and SKI protein bind together and repress the Rorγt promoter, thus when *Smad4* gene is deleted from CD4^+^ T cells, IL-6 can induce T_H_17 cells without TGF-β^2^. Interestingly, in embryonic stem cells, Smad2 and Smad3 complex cannot access the target genes without TIF1-γ^20^. Therefore, TIF1-γ is considered an alternative pathway to Smad4. Indeed, Smad2/Smad3-TIF1-γ complex controls the differentiation of hematopoietic stem/progenitor cells in response to TGF-β, while Smad2/3-Smad4 complex regulates the proliferation of those cells^21^. Recent data suggest that the deletion of *Tif1g* in CD4^+^ T cells decreases the expression of IL-17A and increases IL-10 while the cells are differentiating into T_H_17 cells^22^. Of note, this latter study is performed either in vitro or it addresses the role of TIF1-γ during the differentiation of CD4^+^ T cells into T_H_17 cells, rather than in vivo or on mature T_H_17 cells. Therefore, the role of TGF-β on mature T_H_17 cells and through which pathways TGF-β signals, are still unclear.

In this study, we find that the anti-inflammatory fate of T_H_17 cells contributes to maintaining intestinal immune homeostasis. In addition, our data show that the anti-inflammatory fate of T_H_17 cells impairs an effective immune response to *S*. *aureus*, but is essential to control the resolution of the intestinal inflammation. Furthermore, we show that all this is orchestrated by TGF-β via Smad3 and Smad4 signalling and not via TIF1-γ. Finally, we extended part of these findings to human biology. Together, these observations broaden our understanding about how late fate decision of T_H_17 cells is controlled and about the physiological implications behind T_H_17 plasticity.

## Results

### T_H_17 derived IL-10 contributes to intestinal homeostasis

**Fig. 1:**
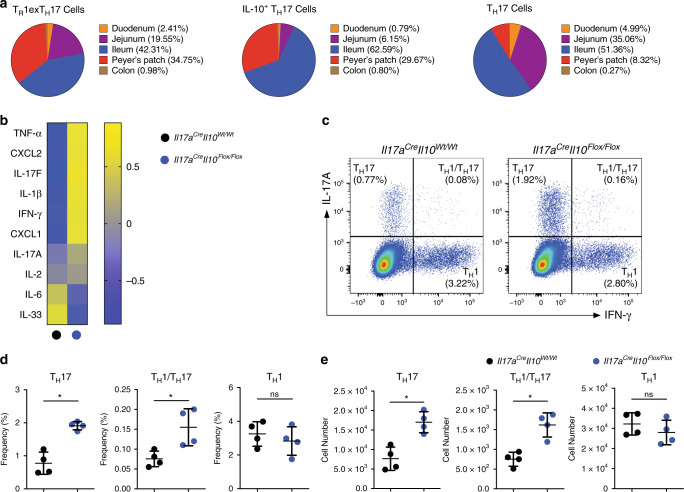
T_H_17 derived IL-10 contributes to intestinal homeostasis. **a** Tissue distribution of the indicated cell populations within the intestine. Cells were isolated from indicated intestinal tracts of the Fate^+^ mice. All cell populations are pre-gated on Foxp3^−^, YFP^+^, CD4^+^ T cells and then described as T_R_1^exTH17^ (IL-10^eGFP+^ IL17A^Kata−^), IL-10^+^ T_H_17 (IL-10^eGFP+^ IL17A^Kata+^) and T_H_17 (IL-10^eGFP–^ IL17A^Kata+^) cells on the basis of the reporter molecules. Cell numbers from three cumulative experiments are used to calculate mean percentage values of the indicated cell populations in different intestinal compartments. **b** Heatmap showing normalized mRNA expression value (Z-score) of different cytokines/chemokines in small intestinal tissues. **c**. Flow cytometric analysis of small intestinal CD4^+^ T cells isolated from the indicated mouse lines under steady state. Intracellular staining for both IL-17A and IFN-γ was then performed to identify T_H_17 (IL-17A^+^ IFN-γ^−^), T_H_1/T_H_17 (IL-17A^+^ IFN-γ^+^) and T_H_1 (IL-17A^−^ IFN-γ^+^) cells. A pre-gate on CD4^+^ T cells is applied. **d**, **e** Statistical analysis of frequencies (**d**) and numbers (**e**) are reported. One representative experiment out of three is shown. Each dot represents one mouse (*n*_wild type_ = 4, *n*_KO_ = 4). Mean ± S.D.; ns, not significant; *P < 0.05 by Mann–Whitney *U* test. Source data are provided as a Source data file.

We previously showed that some T_H_17 cells can express IL-10 (IL-10^+^ T_H_17) and eventually fully convert into bona fide T_R_1 cells (T_R_1^exTH17^): the anti-inflammatory fate of T_H_17 cells^6,7^. Of note, the identity of these T_R_1^exTH17^ cells has been previously shown at both the transcriptional and functional levels^7^. However, the spatial distribution of these different cell statuses (i.e. IL-10^+^ T_H_17 and T_R_1^exTH17^ cells) remained to be defined. We used the Fate^+^ (*Il17a*^*Cre*/*Katushka*^, *Rosa26*-*STOP*^*Flox*/*Flox*^ *YFP* (*R26*^*YFP*^), *Il10*^*eGFP*^, *Foxp3*^*RFP*^) mice and assessed the presence of T_H_17 (Foxp3^RFP−^ IL-17A^*Katushka+*^ IL-10^eGFP−^ R26^YFP+^), IL-10^+^ T_H_17 cells (Foxp3^RFP−^ IL-17A^*Katushka+*^ IL-10^eGFP+^ R26^YFP+^) and T_R_1^exTH17^ cells (Foxp3^RFP−^ IL-17A^*Katushka*−^ IL-10^eGFP+^ R26^YFP+^) in lymphoid and in intestinal tissues. We observed that the majority of these two T_H_17-derived IL-10 producing cell populations (i.e. IL-10^+^ T_H_17 cells and T_R_1^exTH17^ cells) reside in the Peyer’s Patches (PP) and in the ileum (Fig. 1a). Considering that the small intestine and PP host several other types of anti-inflammatory cells, such as Foxp3^+^ Treg and T_R_1 cells, which also secrete high levels of IL-10^23–25^, we wondered whether the anti-inflammatory fate of T_H_17 cells is redundant for the intestinal homeostasis. To this end, we crossed *Il17a*^Cre^ mice with *Il10*^*Flox*/*Flox*^ and *R26*^*YFP*^ mice (from here on referred to as *Il17a*^Cre^ *Il10*^*Flox*/*Flox*^) (Supplementary Fig. 1a). First, we confirmed the efficiency of IL-10 deletion in T_H_17 cells in vivo and in vitro (Supplementary Fig. 1b–e). Then we investigated whether intestinal homeostasis in these mice was altered. Although we did not observe any obvious intestinal tissue damage microscopically (Supplementary Fig. 1f), many pro-inflammatory genes such as *Il1b*, *Il17f*, *Cxcl1*, *Ifng and Tnfa* are elevated in the intestinal tissues of *Il17a*^Cre^ *Il10*^*Flox*/*Flox*^ mice, compared to their littermate control mice (*Il17a*^Cre^ *Il10*^*Wt*/*Wt*^) under steady state conditions (Fig. 1b). Furthermore, a more detailed cellular analysis revealed that under steady state conditions, there was an accumulation of T_H_17 and T_H_1/T_H_17 cells in the small intestine of the *Il17a*^Cre^ *Il10*^*Flox*/*Flox*^ mice compared to their wild-type littermate controls. Of note, the T_H_1 cell population was comparable in frequency and number between the *Il17a*^Cre^ *Il10*^*Flox*/*Flox*^ and control mice, suggesting that T_H_17 cell derived IL-10 has a specific capacity to regulate T_H_17 cell expansion in the intestine (Fig. 1c–e).

Next, we profiled the IL-10 expression in different types of immune cells to assess the different potential contribution to the phenotype observed in the *Il17a*^Cre^ *Il10*^*Flox*/*Flox*^ mice. We observed that more than 90% of the cells that co-express IL-10 and YFP (indicating IL-17A production) in the small intestine are CD4^+^ T cells (Supplementary Fig. 1g).

Finally, we tested whether *Il17a*^Cre^ *Il10*^*Flox*/*Flox*^ mice acquired an extra intestinal spontaneous immune dysregulation, but we could not observe any immune abnormality in the thymus, spleen and other peripheral lymphoid organs (Supplementary Fig. 2).

These data reveal the distribution of the IL-10^+^ T_H_17 cells and T_R_1^exTH17^ cells along the small intestinal tract. Moreover, these data suggest that the anti-inflammatory fate of T_H_17 cells plays a non-redundant role in maintaining the cellular and molecular immune homeostasis in the small intestine.

### IL-10 deletion in T_H_17 enhances antibacterial immunity

We next hypothesized that IL-10 deletion in IL-17A producing cells may lead to more efficient immunity at the expense of immunological tolerance. To test this hypothesis, we first used a *S. aureus* infection mouse model and then an intestinal inflammation mouse model followed by a spontaneous resolution phase.

**Fig. 2:**
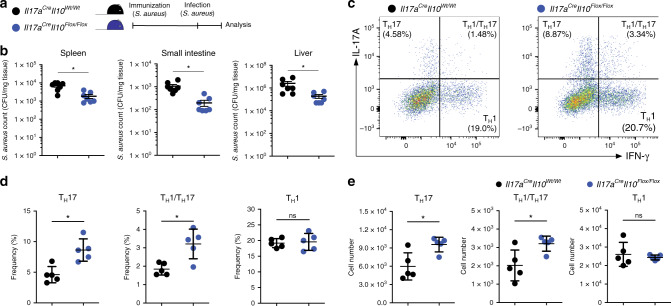
IL-10 deletion in T_H_17 enhances antibacterial immunity. T_H_17 derived IL-10 impairs the development of an efficient immunity against *S. aureus*. **a** A schematic depiction of the experimental plan. **b** Colony-forming units (CFUs) of *S. aureus* from different organs. Data are cumulative of two independent experiments. Each dot represents one mouse (*n*_wild type_ = 7, *n*_KO_ = 7). Mean ± SEM.; *P < 0.05 by Mann–Whitney *U* test. **c** Flow cytometric analysis of small intestinal CD4^+^ T cells after *S. aureus* infection. Small intestinal lymphocytes were isolated from the indicated mouse lines and intracellular staining for both IL-17A and IFN-γ was performed to identify T_H_17 (IL-17A^+^ IFN-γ^−^), T_H_1/T_H_17 (IL-17A^+^ IFN-γ^+^) and T_H_1 (IL-17A^−^ IFN-γ^+^) cells. A pre-gate on CD4^+^ T cell is applied. One representative experiment out of three is shown. **d**, **e** Statistical analysis of frequencies (**d**) as well as numbers (**e**) are reported. One representative experiment out of three is shown. Each dot represents one mouse (*n*_wild type_ = 5, *n*_KO_ = 5). Mean ± S.D.; ns, not significant; *P < 0.05 by Mann–Whitney *U* test. Source data are provided as a Source data file.

We and others have previously shown that intravenous infection of *S. aureus* promotes the accumulation and activation of IL-10-producing T_H_17 cells at the intestinal barrier^6,7,26^. Mechanistically we showed that the superantigen of *S. aureus*, staphylococcus aureus enterotoxin B (SEB), induces a cytokine storm and rapid induction/expansion of intestinal T_H_17 cells^6^. Here we first immunized the *Il17a*^Cre^ *Il10*^*Flox*/*Flox*^ mice with heat killed *S. aureus* and then infected them to test the efficiency of bacterial clearance (Fig. 2a). We observed that the *Il17a*^Cre^ *Il10*^*Flox*/*Flox*^ mice had a lower bacterial burden compared to control mice (*Il17a*^Cre^ *Il10*^*Wt*/*Wt*^) in the spleen, small intestine and the liver (Fig. 2b). We also confirmed the frequency and the number of both T_H_17 and T_H_1/T_H_17 in *Il17a*^Cre^ *Il10*^*Flox*/*Flox*^ mice were increased after treatment, as already observed under steady state conditions (Fig. 2c–e). These results suggest that IL-10 deletion in IL-17A producing cells leads to a more efficient immune response against *S*. *aureus*.

### IL-10 deletion in T_H_17 impairs intestinal immune regulation

**Fig. 3:**
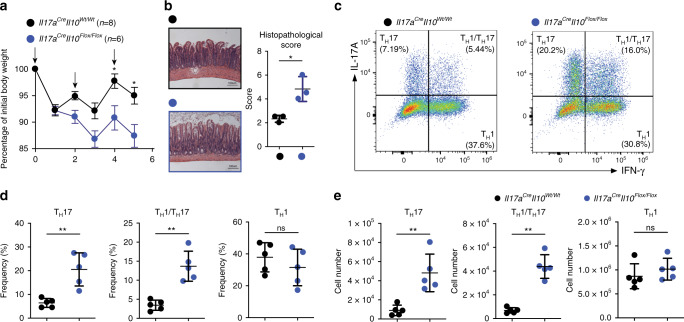
IL-10 deletion in T_H_17 impairs intestinal immune regulation. **a** Percentage of initial body weight after anti-CD3 mAb. The arrows indicate the injection of anti-CD3 mAb. Data are cumulative of three independent experiments. Mean ± SEM.; *P < 0.05, by two-way ANOVA with Bonferroni’s post test. **b** Representative histological pictures of H&E stained small intestines after anti-CD3 mAb treatment. For the statistical analysis on the right, one representative experiment out of two is shown. Each dot represents one mouse (*n*_wild type_ = 3, *n*_KO_ = 3). Mean ± S.D.; *P < 0.05 by Welch’s *t*-test. **c** Flow cytometric analysis of small intestinal CD4^+^ T cells after anti-CD3 mAb treatment. Small intestinal lymphocytes were isolated from the indicated mouse lines and intracellular staining for both IL-17A and IFN-γ was performed to identify T_H_17 (IL-17A^+^ IFN-γ^−^), T_H_1/T_H_17 (IL-17A^+^ IFN-γ^+^) and T_H_1 (IL-17A^−^ IFN-γ^+^) cells. A pre-gate on CD4^+^ cell is applied. **d**, **e** Statistical analysis of frequencies (**d**) and numbers (**e**) are shown. One representative experiment out of three is shown. Each dot represents one mouse (*n*_wild type_ = 5, *n*_KO_ = 5). Mean ± S.D.; ns, not significant; **P < 0.01 by Mann–Whitney *U* test. Source data are provided as a Source data file.

Next, we tested whether IL-17A-producing cell derived IL-10 plays an essential role during the resolution of inflammation in the small intestine. Thus, we challenged mice with anti-CD3 monoclonal antibody (mAb) treatment, a mouse model that we and others have previously established to induce a transient inflammation in the small intestine followed by the re-establishment of immunological tolerance^6,7,25,27,28^. We found that *Il17a*^Cre^ *Il10*^*Flox*/*Flox*^ mice were more susceptible to inflammation induced weight loss compared to control mice (*Il17a*^Cre^ *Il10*^*Wt*/*Wt*^) (Fig. 3a). Furthermore, the small intestines of the *Il17a*^Cre^ *Il10*^*Flox*/*Flox*^ mice showed more severe tissue damage, represented by disrupted villi structures and edema compared to control mice (Fig. 3b). In addition, we observed a selective accumulation of T_H_17 cells and T_H_17/T_H_1 cells in the intestine of the *Il17a*^Cre^ *Il10*^*Flox*/*Flox*^ mice (Fig. 3c–e). We next questioned whether a different expression of IL-10R among the T_H_17, T_H_17/T_H_1 and T_H_1 cells could justify the selective accumulation of T_H_17 cells and T_H_17/ T_H_1 but not of T_H_1 cells in the *Il17a*^Cre^ *Il10*^*Flox*/*Flox*^ mice. In line with what we published previously^25^, we indeed observed that T_H_17 and T_H_17/T_H_1 cells express higher levels of IL-10R compared to T_H_1 cells (Supplementary Fig. 3a, b).

Collectively, these data indicate that the lack of IL-10 expression in IL-17A producing cells promotes immunity against *S*. *aureus* infection, but leads to impaired resolution of the immune response.

### TGF-βRII is required for mature T_H_17 cells to produce IL-10

**Fig. 4:**
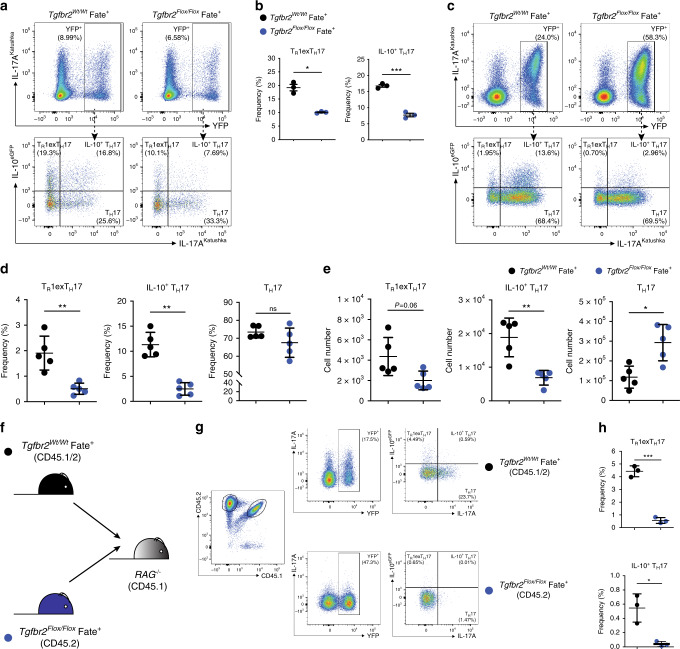
TGF-βRII is required for mature T_H_17 cells to produce IL-10. **a** Flow cytometric analysis of in vitro cultured total CD4^+^ T cells. Cells were polarized under T_H_17 condition for 5 days. A pre-gate on viable (DAPI^−^), CD4^+^ T cell population is applied. **b** Statistical analysis of IL-10^+^ T_H_17 and T_R_1^exTH17^ cells is shown by one representative experiment out of three. Each dot represents one mouse (*n*_wild type_ = 3, *n*_KO_ = 5). Mean ± S.D.; *P < 0.05, ***P < 0.001 by Welch’s *t*-test. **c** Flow cytometric analysis of small intestinal CD4^+^ T cells after anti-CD3 mAb treatment. The indicated reporter mouse lines were used. Top panels are pre-gated on Foxp3^−^ CD4^+^ T cells. Bottom panels are gated on YFP^+^ cells, as shown, and the indicated populations are identified on the basis of the reporter molecules as follows: T_R_1^exTH17^ cells: IL-10^eGFP+^ IL-17a^Kata−^; IL-10^+ ^T_H_17 cells: IL-10^eGFP+^ IL-17a^Kata+^; T_H_17 cells: IL-10^eGFP–^ IL-17a^Kata+^. **d**, **e** Frequencies (**d**) and numbers (**e**) of the indicated populations are shown. One representative experiment out of three is shown. Each dot represents one mouse (*n*_wild type_ = 5, *n*_KO_ = 5). Mean ± S.D.; ns, not significant; *P < 0.05, **P < 0.01 by Mann–Whitney U test. **f**. Experimental design for Co-transfer of total CD4^+^ T cells isolated from wild-type Fate^+^ mouse and *Tgfbr2*^*Flox/Flox*^ Fate^+^ mouse lines. **g**. Flow cytometric analysis of small intestinal co-transferred CD4^+^ T cells after anti-CD3 mAb treatment. Small intestinal lymphocytes were isolated from the indicated mouse lines and intracellular staining was then performed to identify the indicated cell populations in combination with reporter molecules for IL-10 (eGFP) and IL-17a lineage (YFP). All cell populations are pre-gated on CD4^+^ T cells. **h** Frequencies of T_R_1^exTH17^ and IL-10^+^ T_H_17 in G is shown by one representative experiment out of two. Each dot represents one recipient mouse (*n* = 3). Mean ± S.D.; *P < 0.05, ***P < 0.001 by Welch’s *t*-test. Source data are provided as a Source data file.

Despite the fact that the molecular signals inducing IL-10 in naïve CD4^+^ T cells have been largely explored, the molecular mechanisms that control IL-10 production in mature T_H_17 cells in vivo remain unknown. TGF-β is an obvious candidate considering its effect on naive CD4^+^ T cells. To this end, we first differentiated T_H_17 cells in vitro, FACS sorted IL-17A producing CD4^+^ T cells and then re-stimulated these cells in the presence of increasing doses of TGF-β. These results confirmed our previous findings^7^ and further showed that TGF-β promotes IL-10 production also in mature T_H_17 cells in a concentration-dependent manner (Supplementary Fig. 4a–d). However, this was an in vitro experiment and whether this is also the case in vivo remained to be tested. Therefore, we tested the role of TGF-β on mature T_H_17 cells using a loss-of-function approach. To this end, we crossed *Tgfbr2*^*Flox*/*Flox*^ with the Fate^+^ mice (Supplementary Fig. 5a). This newly generated *Tgfbr2*^*Flox*/*Flox*^ Fate^+^ mouse model allowed us to specifically and selectively delete *Tgfbr2* gene in IL-17A-producing cells, and in the meantime to track the T_H_17 cell fate as well as cytokine profile. We first confirmed that the deletion of *Tgfbr2* occurs in a T_H_17-specific manner in *Tgfbr2*^*Flox*/*Flox*^ Fate^+^ (Supplementary Fig. 5b). Next, we tested whether *Tgfbr2*-deficient T_H_17 cells have any impairment in up-regulating IL-10 expression when cultured in vitro. We found that both the IL-10^+^ T_H_17 population and T_R_1^exTH17^ population were reduced in cultured CD4^+^ T cells isolated from *Tgfbr2*^*Flox*/*Flox*^ Fate^+^ mice compared to CD4^+^ T cells isolated from littermate controls (Fig. 4a, b). Of note, we didn’t observe an obvious defect of primary T_H_17 cell differentiation with *Tgfbr2*^*Flox*/*Flox*^ Fate^+^ cells (Supplementary Fig. 5c, d), which is in line with the previous observation that IL-17A^Cre^-mediated gene deletion did not affect primary T_H_17 cell proliferation and differentiation^29^.

Finally, we examined whether T_H_17 cells require TGF-βRII in order to produce IL-10 in vivo. We observed that under steady state conditions, intestinal CD4^+^ T cells marked by YFP (indicating *Tgfbr2* deletion) from *Tgfbr2*^*Flox*/*Flox*^ Fate^+^ mice had an obvious impairment in IL-10 and IL-17A production compared to those cells isolated from *Tgfbr2*^*Wt*/*Wt*^ Fate^+^ mice (Supplementary Fig. 5e–g). Since TGF-β promotes the expression of Foxp3, as a control we tested whether the small expression of Foxp3^+^ among the intestinal R26^YFP+^ CD4^+^ T cells was dysregulated in the *Tgfbr2*^*Flox/Flox*^ Fate^+^ mice. However, we did not observe any statistically significant dysregulation of Foxp3 and more importantly no difference in the number of Foxp3^+^ YFP^+^ cells between the *Tgfbr2*^*Flox/Flox*^ Fate^+^ and *Tgfbr2*^*Wt*/*Wt*^ Fate^+^ mice (Supplementary Fig. 5h, i).

We then injected anti-CD3 mAb to induce a transient intestinal inflammation in the small intestine of these mice. Similar to what we observed by using the *Il17a*^Cre^ *Il10*^*Flox*/*Flox*^ mice, *Tgfbr2*^*Flox*/*Flox*^ Fate^+^ were more susceptible to intestinal inflammation compared to the relative control. The *Tgfbr2*^*Flox/Flox*^ Fate^+^ lost more weight and had a more severe intestinal pathology than control *Tgfbr2*^*Wt*/*Wt*^ Fate^+^ mice (Supplementary Fig. 6a, b). We then harvested the intestinal tissues and analysed the lymphocyte composition. We observed that both the frequency and the amount of IL-10^+^ T_H_17 cells and T_R_1^exTH17^ cells from the intestines of *Tgfbr2*^*Flox*/*Flox*^ Fate^+^ mice were significantly lower than those from WT Fate^+^ mice (Fig. 4c–e). Of note, similar to what is seen under steady state conditions, we did not observe statistically significant changes in the expression of Foxp3 among R26^YFP+^ CD4^+^ T, nor did we observe differences in the number of Foxp3^+^ R26^YFP+^ cells between the *Tgfbr2*^*Flox/Flox*^ Fate^+^ and *Tgfbr2*^*Wt*/*Wt*^ Fate^+^ mice (Supplementary Fig. 6c, d).

To exclude the possibility that reduced IL-10 production from the CD4^+^ YFP^+^ cells of *Tgfbr2*^*Flox*/*Flox*^ Fate^+^ mice was a consequence of more severe inflammation, we co-transferred CD4^+^ T cells from both CD45.2 *Tgfbr2*^*Flox*/*Flox*^ Fate^+^ mice and CD45.1/2 *Tgfbr2*^*Wt*/*Wt*^ Fate^+^ mice into *Rag1*^−/−^ mice. After we engrafted the cells, we injected anti-CD3 mAb and finally harvested the cells. We found that even when the cells resided in the same environment, *Tgfbr2*-deficient T_H_17 cells lost their ability to express IL-10 compared to wild-type cells, which still produced IL-10 (Fig. 4f–h). We also observed that *Tgfbr2*-deficient R26^YFP+^ T_H_17 cells expanded and failed to preserve the expression of IL-17A (Supplementary Fig. 6e), suggesting a role of TGF-β signalling in controlling proliferation and IL-17A maintenance in this particular experimental setting. Taken together, these data indicate that mature T_H_17 cells intrinsically require signalling via TGF-βRII to promote the expression of IL-10 in vitro and in vivo.

### TGF-β signals via Smad3/4 to regulate IL-10 in T_H_17 cells

The next step was to explore the signalling pathway that drives IL-10 expression upon TGF-β engagement with its receptor on mature T_H_17 cells. After TGF-β engages its receptor, Smad2 and Smad3 complex is phosphorylated and by binding to either TIF1-γ or Smad4, translocates into the nucleus to activate gene expression^21^. Whether either Smad4 or TIF1-γ controls TGF-β-mediated IL-10 expression in mature T_H_17 cells remained to be tested.

We first examined the role of TIF1-γ in the expression of IL-10 in T_H_17 cells. To this end, we made *Tif1g*^*Flox*/*Flox*^ Fate^+^ mice, which similar to the *Tgfbr2*^*Flox*/*Flox*^ Fate^+^ mice described above, in order to specifically deplete *Tif1g* in *Il17a* producing cells (Supplementary Fig. 7a). After inducing intestinal inflammation in these mice, we observed that both IL-10^+^ T_H_17 cells and T_R_1^exTH17^ cells remain comparable between *Tif1g*^*Flox*/*Flox*^ Fate^+^ mice and their wild-type littermate controls, suggesting that TIF1-γ is not required for IL-10 production in T_H_17 cells in vivo (Supplementary Fig. 7b–d).

**Fig. 5:**
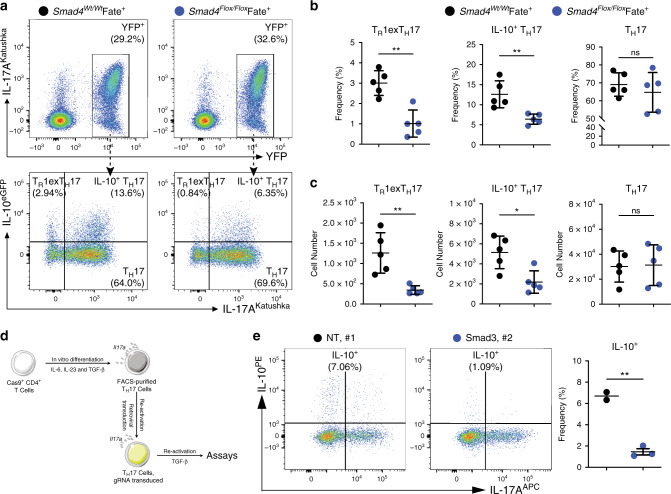
TGF-β signals via Smad3/4 to regulate IL-10 in T_H_17 cells. **a** Flow cytometric analysis of small intestinal CD4^+^ T cells after anti-CD3 mAb. The indicated reporter mouse lines were used. Top panels are pre-gated on Foxp3^−^, CD4^+^ T cells. Bottom panels are pre-gated on YFP^+^ cells, and the indicated populations are identified on the basis of the reporter molecules as follows: T_R_1^exTH17^ cells: IL-10^eGFP+^ IL-17a^Kata−^; IL-10^+^ T_H_17 cells: IL-10^eGFP+^ IL-17a^Kata+^; T_H_17 cells: IL-10^eGFP−^ IL-17a^Kata+^. **b**, **c** Frequencies (**b**) and numbers (**c**) of T_R_1^exTH17^, IL-10^+^ T_H_17 and T_H_17 cells in A are one representative experiment out of three. Each dot represents one mouse (*n*_wild type_ = 5, *n*_KO_ = 5). Mean ± S.D.; ns, not significant; *P < 0.05, **P < 0.01 by Mann-–Whitney *U* test. **d** Experimental design of knocking out Smad3 in in vitro differentiated mature T_H_17 cells by using CRISPR/Cas9 technology. **e** Flow cytometric analysis of in vitro cultured mature T_H_17 cells after knocking out Smad3 by using gRNAs targeting Smad3. Cells were pre-gated on viable CD4^+^ T cells. NT, non-targeting control. Each dot represents an individual gRNA (*n*_NT_ = 2, *n*_Smad3_ = 3). Mean ± S.D.; **P < 0.01 by Welch’s *t*-test. Source data are provided as a Source data file.

We then evaluated whether Smad4 is important for TGF-β-mediated IL-10 expression. To address this, we first created *Smad4*^*Flox*/*Flox*^ Fate^+^ mice in order to delete *Smad4* in T_H_17 cells (Supplementary Fig. 8a). *Smad4*-deficient CD4^+^ T cells had a slight, but significant impairment in IL-10 production when cultured under T_H_17 conditions in vitro (Supplementary Fig. 8b, c). These mice were then injected with anti-CD3 mAb and IL-10^+^ T_H_17 and T_R_1^exTH17^ cell populations were analysed. In line with the in vitro observations, both types of cells were reduced in the small intestines of *Smad4*^*Flox*/*Flox*^ Fate^+^ mice compared with the wild-type control mice (Fig. 5a–c).

Finally, we also determined the role of Smad3 in regulating IL-10 production in T_H_17 cells. We disrupted the function of Smad3 by using either Crispr/Cas9 mediated gene depletion (Fig. 5d, e), or small chemical inhibitor SIS3 in in vitro differentiated T_H_17 cells (Supplementary Fig. 9a, b). In both cases, we observed that the induction of IL-10 in in vitro differentiated T_H_17 cells by TGF-β was impaired when Smad3 was deleted/blocked. These data suggest that Smad3 is also required for TGF-β induced IL-10 production in T_H_17 cells in vitro.

Taken together, these data show that while TIF1-γ is dispensable, Smad4 and Smad3 are necessary for TGF-β mediated IL-10 induction in mature T_H_17 cells.

### Smad3 and Smad4 activate *Il10* transcription in T_H_17 cells

**Fig. 6:**
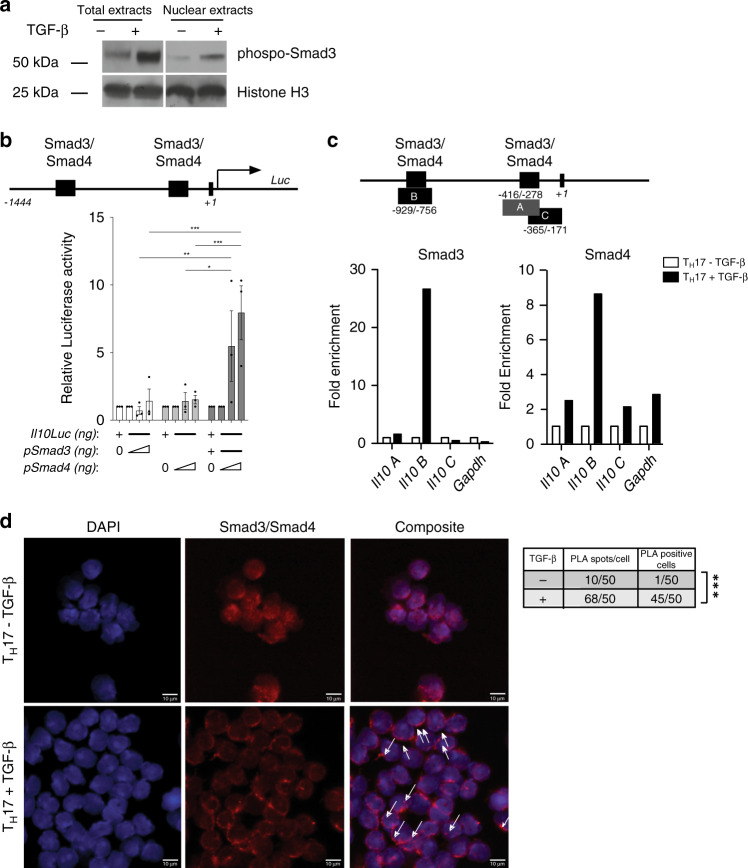
Smad3 and Smad4 activate *Il10* transcription in T_H_17 cells. **a** Western blot of T_H_17 cell-total/nuclear extracts challenged with or without TGF-β for 30 min. One representative experiment out of two is shown. **b** Schematic representation of the proximal murine *Il10* promoter (from −1444 to +1 bp) driving the expression of the luciferase reporter gene (*luc*). The predicted binding sites for Smad3 and Smad4 as well as the *Il10* TSS are depicted (top). Relative Luc activity driven by Smad3 and Smad4 on proximal *Il10* promoter (bottom). Mean ± SEM (*n* = 3). *P < 0.05, **P < 0.01, and ***P < 0.001 by ordinary two-way ANOVA and Tukey’s multiple comparisons test. **c** Schematic representation of the proximal murine *Il10* promoter (from −1444 to +1 bp) indicating the predicted binding sites for Smad3 and Smad4 and the depicted amplicons A (from −416 to −278 bp), B (from −929 to −756 bp), and C (from −365 to −171 bp) (top) that were used to amplify the chromatin Immunoprecipitated DNA. Smad3 and Smad4 Chromatin immunoprecipitation (ChIP) of sorted T_H_17 cells challenged with or without TGF-β (bottom). One representative experiment out of three is shown. **d** Proximity Ligation Assay (PLA) using sorted T_H_17 cells challenged with or without TGF-β. White arrows indicate positive Smad3/Smad4 interaction signal (red dots) superimposed on nuclei stained with DAPI (blue). Scale bar represents 10 μm. Number of PLA spots per cell and the number of PLA positive cells are reported in the box. One representative experiment out of two is shown. ***P < 0.001 for comparison by *χ*^2^ test. Source data are provided as a Source data file.

We next sought to test whether Smad3 and Smad4 bind to the *Il10* promoter in mature T_H_17 cells. First, we tested whether phospho-Smad3 translocates into the nucleus shortly after TGF-β stimulation. Nuclear extracts of T_H_17 cells were immunoblotted and we observed phospho-Smad3 in the nuclei of these cells (Fig. 6a). Next, we tested whether Smad3 and Smad4 are able to induce *Il10* gene transcription. We first cloned *Il10* proximal promoter (−1444 to +1) in the pGL2 vector upstream of *Luciferase* reporter gene, and *Smad3* and *Smad4* genes in pCMV and pCMVβ mammalian expression vectors respectively. Co-transfection of *Il10* proximal promoter with increasing concentrations of either only Smad3 or only Smad4 expression plasmids did not result in significant upregulation of luciferase activity. However, when increasing concentrations of Smad4 plasmid were co-transfected with a constant amount of Smad3 plasmid, a significant increase in *Il10*-luciferase activity was observed (Fig. 6b). These data suggest that Smad3 and Smad4 synergistically activate the *Il10* promoter.

In order to show that Smad3 and Smad4 directly bind the *Il10* promoter in mature T_H_17 cells, we performed Chromatin Immunoprecipitation (ChIP) experiments with FACS sorted T_H_17 cells stimulated with or without TGF-β for 30 min. We found that 30 min after TGF-β treatment, both Smad3 and Smad4 accessed and bound the *Il10* promoter on the −929/−756 element. Of note, between −929/−756 and −286/−283 base pairs on *Il10* promoter lie predicted Smad3/4 binding sites (Fig. 6c). As a negative control, we interrogated the GAPDH TATA box promoter region where Smad3 and Smad4 proteins do not bind, and as expected we did not see any signal. Finally, in order to show that Smad3 and Smad4 proteins form a complex in T_H_17 cells, we performed Proximity Ligation Assay (PLA) in FACS sorted T_H_17 challenged for 30 min with TGF-β, using a plus and a minus ligation probe specific for anti-mouse Smad3 and anti-rabbit Smad4 antibodies, respectively. Confocal microscopic images reveal that the Smad3/4 complexes are only visible in the nucleus of T_H_17 cells challenged with TGF-β (Fig. 6d). Interestingly, this protein complex forms 3–5 bright, closely spaced foci per cell. The PLA spots were located in the rim of the T_H_17 cell nucleus associated with the nuclear membrane. Taken together, these results show that Smad3 and Smad4 proteins are instructed by TGF-β signal, form a nuclear complex that bind to the proximal *Il10* promoter and transactivates *Il10* transcription in T_H_17 cells.

### TGF-β promotes IL-10 expression in human T_H_17 cells

**Fig. 7:**
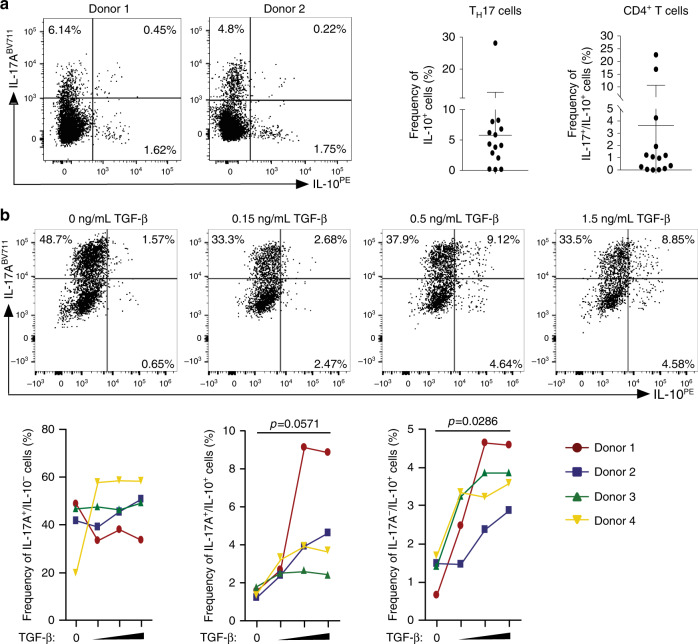
TGF-β promotes IL-10 expression in human T_H_17 cells. **a** Two representative dot plots of CD4^+^ T cells extracted from healthy human colonic tissue, stained intracellularly for IL-17A and IL-10. The diagrams show the frequencies of IL-17A^+^/IL-10^+^ within the T_H_17 cells (left) and within the total CD4^+^ T cell (right) (*n* = 14) **b** Representative dot plots of T_H_17 cells isolated from human blood donor, stimulated and cultured under increasing TGF-β1 concentrations for 5 days and finally stained for IL-17A and IL-10 intracellularly. The graphs below show the percentages of IL-17A^+^/IL-10^−^, IL-17A^+^/IL-10^+^ and IL-17A^−^/IL-10^+^ cells from four different blood donors. *P* values were calculated between the lowest TGF-β and highest TGF-β concentration conditions by Mann–Whitney *U* test (*n* = 4). Source data are provided as a Source data file.

We tested whether human T_H_17 cells isolated from the intestine can also produce IL-10. We observed that a small but consistent frequency of CD4^+^ T cells isolated from healthy colon tissues from 14 human donors, co-produce IL-17A and IL-10 (Fig. 7a). Next, we wondered whether human T_H_17 cells can also acquire the expression of IL-10 when stimulated with TGF-β. We therefore isolated circulating CD45RA^low^ CD25^−^ IL-17A^+^ (T_H_17) cells from human blood using the IL-17A secretion assay and cultured the isolated T_H_17 cells in the presence of increasing TGF-β concentrations. The purity of the T_H_17 cells isolated from human blood was always more than 95% (for a representative purity plot see Supplementary Fig. 10a). Upon examining the CD4^+ ^Foxp3^−^ T cell compartment (see Supplementary Fig. 10b for the gating strategy), we observed that approximately half of the sorted cells, that were formerly IL-17A^+^, ceased to produce IL-17A (i.e. they become exT_H_17 cells) while the other half retained IL-17A expression. However, this phenomenon was not affected by TGF-β (Fig. 7b). In contrast, TGF-β promoted IL-10 expression in both T_H_17 cells and exT_H_17 cells in a concentration-dependent manner (Fig. 7b). Finally, increasing TGF-β concentrations appeared to slightly reduce the frequency and the numbers of IL17A^+^ IFN-γ^+^ and IFN-γ^+^ producing cells (Supplementary Fig. 10c, d). Furthermore, increasing the TGF-β concentrations did not have a reproducible effect on Foxp3 expression in human peripheral blood-isolated T_H_17 cells (Supplementary Fig. 10e). Collectively, these results show that human T_H_17 cells from the intestine and from peripheral blood, are able to produce IL-10 and furthermore, that TGF-β is able to promote dose dependent IL-10 expression in human peripheral blood derived T_H_17 cells.

## Discussion

T_H_17 cells are usually found in the inflamed tissues of IMID patients such as the central nervous system, the kidney, the joints and the heart. However, they are also abundant in the small intestine of healthy individuals without causing any overt diseases^30–32^. The T_H_17 cell plastic behaviour allowing them to acquire both pro- and anti-inflammatory fate can explain such ubiquitous presence and consequences: pathological or physiological. Our data show that it is in fact mainly in the ileum and Peyer’s patches of mice where these cells acquire their anti-inflammatory fate. This suggests that continuous plasticity from T_H_17 cells to IL-10 producing T_H_17 cells and possibly to T_R_1^exTH17^ cells impairs the pathogenic immune reaction of effector T_H_17 cells in the intestinal tract. Supporting this, when T_H_17 cell-derived IL-10 is lacking, the cellular and molecular immune homeostasis of the intestine is disturbed, showing the relevance of the anti-inflammatory fate of these cells. This effect is remarkable considering that only a relatively small fraction of T_H_17 cells acquire an anti-inflammatory function. Finally, our data show that also human T_H_17 cells acquire the regulatory phenotype when exposed to TGF-β1.

Despite the above, we did not observe macroscopic tissue damage in the *Il17a*^Cre^ *Il10*^*Flox/Flox*^ mice. One explanation is the compensatory mechanism which could be exerted by the presence of other regulatory T cells, such as Foxp3^+^ Treg cells and T_R_1 cells able to produce IL-10^23,25^. Indeed, it has to be considered that only a fraction of T_R_1 cells originate from the T_H_17 cells, while the rest originate from other cells, including probably T_H_1 cells^33^. Thus, the latter T_R_1 cell fraction is still functional in this mouse model. Another explanation is the composition of the intestinal microbiota of our mice. It is possible that under different specific pathogen free conditions, also *Il17a*^Cre^ *Il10*^*Flox/Flox*^ mice develop microscopically evident intestinal inflammation.

Interestingly, we observed a specific expansion of intestinal T_H_17 and T_H_1/T_H_17 cells, but not of T_H_1 cells in our *Il17a*^Cre^ *Il10*^*Flox/Flox*^ mouse model. One possible explanation of this cell-selective control mediated by IL-10 is that both T_H_17 and T_H_1/ T_H_17 cells express higher level of IL-10R compared to T_H_1 cells. Alternatively, one could consider that this is due to a dysregulation of Rorgt^+^ Foxp3^+^ Treg cells in *Il17a*^Cre^ *Il10*^*Flox/Flox*^. These cells are indeed known to be able to specifically regulate T_H_17 cells, especially in the colon^34^. However, only a small fraction of *Il17a*^*Cre*^-activated cells are Foxp3^+^ and the large majority of Rorgt^+^ Foxp3^+^ Treg cells are known to express little to no IL-17A and thus are not mainly affected in the small intestine of the *Il17a*^Cre^ *Il10*^*Flox/Flox*^ mice^35,36^. Therefore, these data suggest that a possible dysregulation of Rorgt^+^ Foxp3^+^ Treg cells is not the explanation for the selective increases in the number of T_H_17 cells observed in this study.

T_H_17 cells also play a key role in mediating host defence against bacteria^1^. Through evolution, pathogens have developed mechanisms to evade the immune system of the host. For example, it was shown in mice and humans that *S*. *aureus*, a nosocomial and potentially multi drug resistant bacteria, promotes the anti-inflammatory fate of T_H_17 cells^7,26^. Our data add to this because they show that the T_H_17 cell anti-inflammatory fate can indeed favour dissemination of the pathogens. Given that this anti-inflammatory fate has been preserved throughout evolution, we wondered what its benefit is, and at what cost it comes. Considering that *S*. *aureus* bacteria only becomes a lethal threat when it disseminates through the blood stream, we propose that the benefit is the T_H_17 cell anti-inflammatory fate’s ability to constantly maintain the immune homeostasis. We also propose that the cost of the T_H_17 cell anti-inflammatory fate is rather low, considering that occurrence of a bacterial infection through the blood is relatively unlikely.

The role of TGF-β during the differentiation of T_H_17 cells has been widely studied and also debated^9,37^. It is currently accepted that TGF-β is sufficient, but not necessary for the induction of these cells and in addition, that it favours the development of T_H_17 with regulatory potential. Our experiments aimed to expand the understanding of TGF-β function from the early differentiation phase to the effector phase of T_H_17 cell biology. We showed for the first time directly in vivo that *Tgfbr2*-deficient T_H_17 cells had an impaired capacity to produce IL-10. In addition, the *Tgfbr2*^*Flox/Flox*^ Fate^+^ mice phenocopied the impairment in immune regulation observed in the *Il17a*^Cre^ *Il10*^*Flox/Flox*^ mice. These results broaden the state of knowledge of TGF-β, revealing the role of TGF-β in plasticity and function of T_H_17 cells upon maturation.

Furthermore, we noticed that under steady state conditions and after anti-CD3 mAb treatment, the frequency of YFP^+^ cells increase when TGF-βRII signalling is impaired. This suggests that the expansion of T_H_17 cells, which could otherwise lead to pathogenicity, is controlled by TGF-β. This is in line with the known anti-proliferative effect of TGF-β on CD4^+^ T cells^38^. Furthermore, we observed that the large majority of TGF-βRII deficient YFP^+^ cells lose the expression of IL-17A under steady state conditions and upon transfer into *Rag1*^−/−^ mice, while they maintain the expression of IL-17A during intestinal inflammation. On the one hand, this is in keeping with the concept of a “sufficient but not necessary” role of TGF-β during the differentiation of T_H_17 cells: under certain conditions, TGF-β is necessary to maintain IL-17A expression, but during inflammation it becomes unnecessary. On the other hand, this also suggests that the fraction of T_H_17 cells which do lose IL-17A, but express IL-10 in wild type mice during inflammation have restrained the TGF-β signalling towards Smad3/*Il10* thus avoiding the re-activation of RORγt/IL-17A pathway. It has indeed been shown that TGF-β blocks RORγt activity via Smad3^19^ and that TGF-β can restrain IL-17A by a post-translational deubiquitination of RORγt^28^.

Of note, TGF-β appears to impact IL-10 production also in other effector CD4^+^ T cells such as T_H_1 cells, although the current evidence is controversial. For example, TGF-β has been shown to inhibit IL-10 production in T_H_1 cells by blocking the expression of Blimp-1^39^, while others reported a positive effect of TGF-β on IL-10 production by T_H_1 cells^40,41^. Therefore, TGF-β seems to have different effects on T_H_1 and T_H_17 cells regarding IL-10 production, but further in vivo studies on T_H_1 cells are required to confirm this.

Downstream of TGF-β receptor, our results suggest that the canonical TGF-β mediators Smad3 and Smad4—but not TIF1-γ—promote the expression of IL-10 in mature T_H_17 cells. Apparently in contrast to our data, it has been shown that TIF1-γ blocks the expression of IL-10 in CD4^+^ T cells while differentiating into T_H_17 cells^22^. However, while this study used the *CD4*^*Cre*^ *Tif1g*^*Flox*/*Flox*^ mouse model, we used an *Il17A*^*Cre*^ *Tif1g*^*Flox*/*Flox*^ mouse model instead. One possible explanation is that during T_H_17 cell differentiation, TIF1-γ constrains IL-10 expression by obstructing Smad-recognized *cis*-elements from the Smad4-Smad2/3 complexes, and that this becomes dispensable in mature T_H_17 cells when the *Il10* gene is already accessible. In keeping with the different function of TIF1-γ in the two different phases, this is also evident regarding the regulation of IL-17A expression: TIF1-γ plays a key role in the expression of IL-17A during T_H_17 cell differentiation^22^. However, our data suggest that its deletion in mature T_H_17 cells becomes dispensable for IL-17A expression. Therefore, these datasets are -probably- not incompatible with each other, but rather they reveal different molecular mechanisms operating during the differentiation and maturation phases of T_H_17 cells.

Cell clones of blood derived human T_H_17 cells are capable of acquiring the expression of IL-10 in vitro^26^. In line with this data, here we show that human primary T_H_17 cells are also able to produce IL-10 and are present in the human intestine. It has recently been suggested that IL-27 promotes the expression of IL-10, via the transcription factor c-Maf, in human T_H_17 clones^16^. However, whether IL-27 alone or in combination with TGF-β can also induce the expression of IL-10 in freshly isolated human T_H_17 cells remains to be tested. Nevertheless, our data serve as proof of principle to show that it is possible to turn freshly isolated human T_H_17 cells into IL-10 producer cells.

The transcription factor Blimp1 has also been proposed as being able to promote the conversion of T_H_17 cells into T_R_1 cells^14^. However, it is also known that TGF-β blocks Blimp1 activation^39,42^. Because of this, we did not investigate the role of Blimp1 in our system. However, our data do not exclude a role of Blimp1 in the anti-inflammatory fate of T_H_17 cells and it could be possible that TGF-β and IL-27 are two complementary pathways to induce the conversion of T_H_17 cells into T_R_1 cells in different microenvironments.

Collectively, our data show that the anti-inflammatory fate of T_H_17 cells is not a mere consequence of immune homeostasis, but that it actually plays an indispensable role in the maintenance of this equilibrium. TGF-β mediates the expression of IL-10 via Smad3 and Smad4 and consequently, it is the fundamental environmental signal to tighten the balance between the pro- and anti-inflammatory fates of mature T_H_17 cells. We propose that the T_H_17 cell plasticity between pro and anti-inflammatory fates is a key feature of immune system adaptation and when impaired, immunity and tissue integrity are compromised. Exploring the molecular mechanisms of this biological phenomena will help to identify therapeutic targets, such as Smad3 and Smad4 for new therapies that aim to steer the immune response according to the clinical need, rather than globally suppress it, as the majority of the current therapies have been based upon.

## Methods

### Mice

C57BL/6 *Rag1*^−/−^ mice (Stock #: 002216) and *Rosa26*-*Cas9* knock-in mice (Stock #: 028555) were purchased from the Jackson Laboratories. *Il10*^*Flox*/*Flox*^ mice (kindly provided by Axel Roers) were further crossed with *Il17A*^*Cre*^ mice (kindly provided by Dr. Brigitta Stockinger). *Tgfbr2*^*Flox*/*Flox*^ (purchased from JAX, Stock #: 012603), *Smad4*^*Flox*/*Flox*^ (kindly provided Dr. Elizabeth Robertson^43^) and *Tif1g*^*Flox*/*Flox*^ (kindly provided by Dr. Vesa Kaartinen^44^) mice were further crossed with Fate^+^ mice (*Foxp3*^*RFP*^, *Il10*^*eGFP*^, *Il17A*^*CRE*/*Katushka*^, *R26*^*YFP*^, as described before^7^) to generate *Tgfbr2*^*Flox*/*Flox*^ Fate^+^ (*Tgfbr2*^*Flox*/*Flox*^, *Foxp3*^*RFP*^, *Il10*^*eGFP*^, *Il17A*^*CRE*/*Katushka*^, *R26*^*YFP*^), *Smad4* ^*Flox*/*Flox*^ Fate^+^ (*Smad4*^*Flox*/*Flox*^, *Foxp3*^*RFP*^, *Il10*^*eGFP*^, *Il17A*^*CRE*/*Katushka*^, *R26*^*YFP*^) and *Tif1g* ^*Flox*/*Flox*^ Fate^+^ (*Tif1g*^*Flox*/*Flox*^, *Foxp3*^*RFP*^, *Il10*^*eGFP*^, *Il17A*^*CRE*/*Katushka*^, *R26*^*YFP*^) mice. In these models, cells expressing a high level of *Il17a* activate the CRE recombinase to delete the stop sequence 5′ to YFP and to deplete *Tgfbr2*, *Smad4* or *Tif1g*. Thus, such IL-17A-expressing cells are permanently marked by YFP and in the meantime, lose the expression of *Tgfbr2*, *Smad4* or *Tif1g* genes. All mice were kept under specific pathogen-free (SPF) conditions, on a 12/12 on/off light cycle, maintained at 72 (+/−2) °F with 70% humidity in the animal facility at Yale University. Age- and sex-matched littermates between 8 to 24 weeks of age were used for experiments. Unless they came with special instructions, mice were randomly assigned to different experimental groups and each cage contained animals of all different experimental groups. Both male and female mice were used in experiments. Animal procedures were approved by the Institutional Animal Care and Use Committee (IACUC) of Yale University. Preliminary experiments were tested to determine sample sizes, taking available recourses and ethical use into account.

### Anti-CD3 mAb treatment and intestinal histology

Anti-CD3 mAb (Clone #: 2C11) were injected intra-peritoneally three times (15, 15, and 30 μg per mouse) every other day. Mice were euthanised with CO_2_ 24 h after the last injection. Different parts of small intestines (duodenum, jejunum and ileum) were fixed in Bouin’s Fixative Solutions for 1 h. Tissues were then embedded in paraffin for sectioning. Pictures were taken by optical microscope equipped with camera systems and analysed.

### Lymphocyte isolation from mouse small intestine

Methods were described before in detail^45^. Basically, intestinal tissues were first digested in 1 mM DTE at 37 °C for 30 min to get the IEL fraction. Remaining tissues were then digested in 1 mg/ml collagenase from Clostridium Histolyticum at 37 °C for another 30 min to get the LPL fraction. Both fractions were further applied with Percol gradient centrifugation to enrich lymphocyte populations.

### Flow cytometry antibodies and intracellular cytokine staining

Mouse T cells were stained with monoclonal antibodies to CD4 (GK1.5, Cat # 100428 or RM4-5 Cat # 100536), CD8 (53-6.7 Cat # 100722), NK1.1 (PK136 Cat # 108713), CD19 (6D5 Cat # 115508), CD11b (M1/70 Cat # 101216), CD11c (N418 Cat # 117318), γδTCR (GL3 Cat # 118123), TGFβRII (R&D Systems Cat # FAB532P). All antibodies without further instructions are purchased from eBiolegend and diluted 1:200 for staining at 4 °C. Importantly, TGFβRII staining was performed at 37 °C for 30 min with 1:50 dilution. Although in the figure legends we referred only to CD4^+^ T cells, in each FACS related experiment we specifically analysed CD4^+^ T cells CD8^−^, NK1.1^−^, CD19^−^, CD11b^−^, CD11c^−^, γδTCR^−^. For intracellular cytokine staining, the cells were re-stimulated for 3 h at 37 °C with phorbol 12-myristate 13-acetate (PMA) (Sigma, 50 ng ml^−1^) and ionomycin (Sigma, 1 μg ml^−1^) in the presence of Golgistop (BD Bioscience). Cells were then fixed in 3.7% formaldehyde for 20 min at room temperature. After washing, the cells were then permeabilized in 0.1% NP40 and stained at 37 °C with anti-IL-17A (TC11-18H10.1 Cat # 506925), anti-IFNγ (BD Bioscience, Cat # 554412) antibodies for 30 min with 1:200 dilution. Lymphocytes were re-suspended in PBS, 0.5% FBS, 5 mM EDTA and acquired with an LSRII cytometer (BD Bioscience).

### *Staphylococcus aureus* infections

Heat-inactivated *S. aureus* (ATCC 14458, SEB+ TSST-1-) was injected intravenously (1 × 10^7^ colony forming units per mouse) into 4-week-old mice for immunization purposes. Three weeks later, the mice were injected intravenously again with 1 × 10^6^ live *S. aureus* bacteria. Mice were euthanised with CO_2_ 1 week after the 2nd injection, when they displayed clinical symptoms of sepsis. Different organs were then harvested to examine the colony forming unit (CFU) of *S. aureus*. T cells isolated from intestines were further analyzed by FACS.

### Plasmids and transfections

The coding sequences for Smad3 and Smad4 were cloned via reverse transcription and PCR from murine C57BL/6 splenic leukocytes total RNA with the appropriate primers, Superscript III reverse transcriptase (*Invitrogen*) and Phusion® Hot Start Flex DNA polymerase (*New England Biolabs*). The PCR products were cloned in pGEM®-T Easy vector (Promega) and were subsequently sub-cloned into pCMVT_N_T™ (Promega) and pCMV-Tag2 (Agilent Technologies) mammalian expression vectors respectively, using the appropriate primers and the InFusion ligation system (Takara). The *Il10* proximal promoter (from −1444 to +1 bp) was cloned via PCR from C57BL/6 genomic DNA with appropriate primers and was subcloned into pGL2 vector (Clontech) using the InFusion ligation system (Takara) and appropriate primer sets. Transfections were performed in HEK293T cells with the BES buffered saline/CaCl_2_ method. Briefly, expression and reporter plasmid mixes were left to precipitate for 20 min at room temperature, in a solution containing 0.125 M CaCl_2_, 0.025 M BES, 0.02 NaCl, 0.00075 NaH_2_PO_4_, pH:6.95. The precipitated plasmid mix was overlaid carefully on the HEK293T cell DMEM 10% FBS culture medium. In all, 24 h later the transfection medium was aspirated and fresh DMEM 10% FBS was placed onto the HEK293T cells. Cells were collected 48 h after transfection, washed twice with PBS and lysed in Passive Lysis Buffer (Promega). Luciferase activity was determined using the Luciferase Assay System (Promega) and was measured in a FluoStar Omega reader and Software. Transfection efficiency was determined by spiking all transfections with 0.5 ng pCMV-EGFP vector (Clontech) and measuring GFP activity in the lysates.

### T_H_17 cell differentiation and immunoblot

Briefly, naïve CD4^+^ T cells were isolated from the spleen and lymph nodes of 4–6 mice of the indicated transgenic mouse strains with the mouse naïve CD4^+^ T cell isolation Kit (Stemcell). The cells were cultured for five days under T_H_17 polarizing conditions, in Click’s medium supplemented with 5% FBS, 0,1 mM β-merkaptoethanol, Glutamax™ (Gibco), 100 U/mL Penicillin/Streptomycin, 10 μg/mL anti-CD3, 2 μg/mL anti-CD28, 10 μg/mL anti-IFNγ, 10 μg/mL anti-IL-4, 20 ng/mL murine IL-23, 20 ng/mL murine IL-6, 0.5 ng/mL murine TGF-β1 (unless specially indicated). T_H_17 cells were sorted based on the IL-17A reporter expression resuspended into 2 × 10^6^ cells/mL and then challenged with or without TGF-β for 30′ min. Subsequently the cells were lysed and either total or nuclear extracts were obtained and loaded on 4–15% precast acrylamide gels (Mini-PROTEAN, BIO-RAD). After electrophoresis the proteins were transferred to PVDF membranes. The membranes were immunoblotted with the indicated antibodies and developed according to standard protocols. We loaded the protein extract equivalent corresponding to 8 × 10^5^ T_H_17 cells in each well.

### Retroviral preparation and transduction

For retroviral preparation, 1 μg of individual Smad3 sgRNA vector with 0.5 μg of EcoHelp plasmid were employed to transfect HEK293T cells. In all, 12 h later, media was replaced and virus was grown for another 48 h. Supernatant containing virus was collected and polybrene was added to achieve a final concentration of 4 μg/ml. For retroviral transduction, Cas9-expressing T cells were first activated for 24 h and then spin transduced with viral supernatant.

### Chromatin Immunoprecipitation

Rested T_H_17 cells were challenged or not with 1 μg/mL TGF-β1 for 30 min. The cells were fixed with 1.1% Formaldehyde for 10 min, washed, and re-suspended in 10 mM Tris pH:7.5, 10 mM NaCl, 3 mM MgCl_2_, 1 mM CaCl_2_, 4% NP40 supplemented with protease inhibitors, at a final concentration of 4 × 10^7^ cells/mL. Subsequently, 0.2 units MNase (Sigma) were added for 5 min at 37 °C, followed by 3 mM EGTA and the cells were centrifuged at 500 *g* and re-suspended in 50 mM Hepes pH:7.9, 150 mM NaCl, 5 mM EDTA, 0.5 mM EGTA, 0.1%SDS, 0.1% Na-deoxycholate, supplemented with protease inhibitors and sonicated 3 times for 20 sec. 1% Triton X-100 was added and the samples were centrifuged for 15000 *g* for 15 min at 4 °C. The lysates were extensively dialysed in 10 mM Tris pH: 7.5, 5% glycerol, 1 mM EDTA, 0.5 mM EGTA. For immunoprecipitation, T_H_17 cell lysates were incubated o/n with designated antibodies and the next day were mixed with Dynabeads™ Protein G (Invitrogen, #00374128) in RIPA buffer for 4 h at 4 °C. The beads were washed in low (150 mM NaCl), high (500 mM NaCl) and LiCl (250 mM) salt buffer, the crosslinks were reversed at 65 °C, the samples were purified by Phenol/Chlorophorm extraction, the DNA was precipitated and used in q-PCR reactions.

### Real-time quantitative PCR

qPCR was performed using chromatin immunoprecipitation reactions with the anti-Smad3 and Smad4 and isotype control antibodies, along with the indicated primers and KAPA SYBR FAST qPCR Kit (Kapa Biosystems, Wilmington, MA) in a StepOne cycler (Applied Biosystems, Carlsbad, CA). The CT values from triplicate qPCR reactions were extracted from the StepOne cycler (Applied Biosystems, Carlsbad, CA) onto spreadsheets and were analyzed with the relative quantification method 2^–ΔΔCT^. The abundance level of a given amplicon per sample/condition was determined relative to its input (non-enriched) chromatin abundance and was additionally corrected relative to isotype control antibody immunoprecipitation sample.

### Proximity ligation assay

In vitro differentiated sorted T_H_17 cells were rested for 5 days in Click’s medium supplemented with 2 ng/mL murine IL-2 (PeproTech #212-12) and were subsequently challenged with 1 μg/mL human TGF-β1 for 30 min. Subsequently, the cells were washed, re-suspended in a minimal volume of PBS supplemented with 10% FBS 0.3% BSA, overlaid onto coverslips and were left to dry for 12 h. The cells were rehydrated with PBS, fixed with 4%PFA and permeabilized with 0.3% PBS-Triton X100. After washing, the specimens were blocked, incubated with pospho-Smad3 and Smad4 antibodies and stained with Duolink® In Situ detection Reagents Red (Sigma). After mounting, the slides were analysed by confocal microscopy and the acquired images were opened and processed with Fiji software (Image J).

### Lymphocyte isolation from human intestine

The human colonic tissue is derived from colon cancer patients admitted into the UKE General Surgery Clinic. The study was approved by the Ethical Physicians Committee of Hamburg (Ethik-Kommision der Aertztekammer Hamburg) and Informed Written Consent was obtained from each one of the sample donors. The general characteristics of the participants cohort were five males and nine females with an average age of 69,5 years (±SD = 11 *y*) Healthy tissues adjacent to the tumor, were collected by collaborating surgical personnel, immediately after the completion of the surgical procedure. Next, the tissue samples were processed for lymphocyte isolation. Intraepithelial lymphocytes were isolated by digesting the tissue with Dithiothreitol (DTT Sigma-Aldrich) solution at 37 °C for 20 min, followed by incubation with collagenase IV (100 U; Sigma-Aldrich) for 30 min at 37 °C. The cells were further separated by a Percoll gradient (GE Healthcare). After this the cells were stained for FACS analysis. Only the healthy colonic tissue data analysis with respect to CD4^+^FOXP3^−^IL17A^+^ and IL-10^+^ cell compartment is presented in this manuscript. For a comprehensive description of the patients, please refer to the Source data file of Supplementary Fig. 9e. Part of the human cohort analyzed in this study was also used for assessing the expression of IL-22 in Perez et al.^46^.

### Sorting of humanT_H_17 cells from Buffy Coat

Enriched human Buffy Coat samples retrieved directly from the UKE Transfusion Medicine, were diluted with PBS to a total volume of 350 mL. The diluted blood samples were overlaid to buffered Biocoll separating solution (Merck, #L6715) and centrifuged at 300 g for 20 min. The PBMCs were collected, washed with PBS and stimulated for 3 h with 50 ng/mL PMA and 1μΜ Ionomycin. CD4^+^ T cells were selected with magnetic human CD4 microbeads (#130-045-101, Miltenyi, Bergisch-Gladbach, Germany). IL-17^+^ cells were stained with the IL17A secretion assay detection kit (#130-094-537, Miltenyi, Bergisch-Gladbach Germany) and with human anti-CD4/FITC, anti-CD45RA/Alexa700, anti-CD127/PE.Cy7 and anti-CD25/BV650 and sorted with an Aria Illusion instrument (BD Bioscience). In all, 5–8 × 10^3^ sorted CD4^+^ IL-17A^+^ CD45RA^−^CD127^−^CD25^−^ cells were rested in RPMI medium (supplemented with 10% FBS, 10 mM β-merkaptoethanol, Glutamax, Penicillin/Streptomycin 100 U/mL) plus 2 ng/mL IL-2 (PeproTech). After 48 h the cells were challenged with increasing concentrations of TGF-β1 for five days. Subsequently, cells were stimulated with PMA/Ionomycin, stained for CD4, IL-17A, and IL-10 expression and analysed by FACS.

### Statistics

FACS data were visualized and analyzed with FlowJo 10.5.3 (BD Bioscience). Statistical analyses were calculated in Prism (Graphpad Software). According to the experimental set-up we used Mann–Whitney *U* test or Welch’s *t*-test (Data specified in Figs. 1–5), two-way ANOVA (Fig. 6b) and *χ*^2^ test (Fig. 6d). The *p* values presented in Fig. 7 were calculated via the Mann–Whitney *U* test. The specific statistical analyses used in each figure are further described in the figure legends. When not otherwise specified, the statistical test used is always two-sided. *p* < 0.05 was considered significant.

### Reporting summary

Further information on research design is available in the Nature Research Reporting Summary linked to this article.

## Supplementary information

Supplementary Information

Reporting Summary

## Data Availability

We declare that the data supporting the findings of this study are available within the paper (and its Supplementary Information files). Source data are provided with this paper.

## Source data

Source Data
