# Supplementary material for: The induction and function of the anti-inflammatory fate of TH17 cells
Source: Nat Commun. 2020 Jul 3;11:3334. doi: 10.1038/s41467-020-17097-5 (PMC7335205; doi:10.1038/s41467-020-17097-5)
Supplement: Supplementary file 1 — Supplementary Information [file 41467_2020_17097_MOESM1_ESM.pdf]

Supplementary Information

The induction and function of the anti-inflammatory fate of T_H17 cells

Xu, H., Agalioti, T. *et al.,* 2020

Supplementary Figure 1: Evaluation of T_H17-specific IL-10 depletion mouse model

Supplementary Figure 2: No immune abnormality observed in the periphery

Supplementary Figure 3: T_H17 cells express higher levels of IL-10R compared to T_H1 cells

Supplementary Figure 4: TGF-β promotes IL-10 production in mature T_H17 cells

Supplementary Figure 5: Tgfbr2 is required for T_H17 cells to produce IL-10

Supplementary Figure 6: Tgfbr2 is required for T_H17 cells to produce IL-10 during intestinal inflammation

Supplementary Figure 7: TIF1γ is dispensable for IL-10 production in T_H17 cells

Supplementary Figure 8: Smad4 is required for T_H17 cells to produce IL-10

Supplementary Figure 9: Smad3 is important for IL-10 production in T_H17 cells

Supplementary Figure 10: Human intestinal T_H17 cells produce IL-10; and TGF-β further promotes IL-10 expression

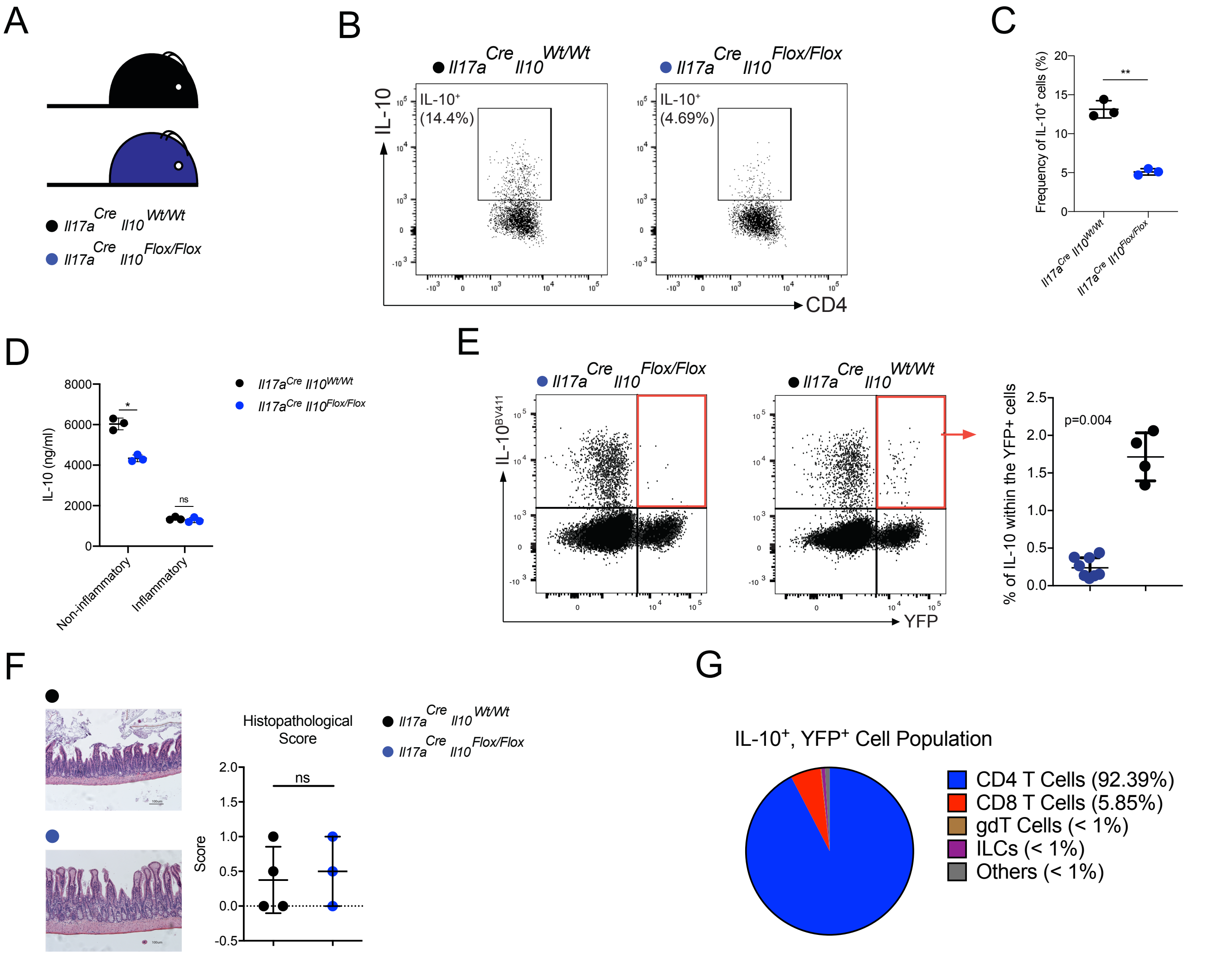

Supplementary Figure 1. Evaluation of T_H17-specific IL-10 depletion mouse model. A. Mouse strains employed to deplete IL-10 production in T_H17 cells. B. Flow cytometric analysis of intestinal lymphocytes isolated from $Il17a^{Cre}$ $Il10^{Wt/Wt}$ as well as $Il17a^{Cre}$ $Il10^{Flox/Flox}$ mice after anti-CD3 treatment. Intracellular staining for IL-10 was then performed. A pre-gate on T_H17 cells ($CD4^+$, $TCR\beta^+$, $IL\text{-}17A^+$) was applied. C. Statistical analysis of frequencies of $IL\text{-}10^+$ cells among T_H17 cells. Each dot represents one mouse ($n_{wild\ type}=3$, $n_{KO}=3$). Mean ± S.D.; **P < 0.01 by Welch's t-test. D. ELISA measurement of IL-10 after *in vitro* culture. Naive $CD4^+$ T cells were isolated from the spleens of $Il17a^{Cre}$ $Il10^{Wt/Wt}$ as well as $Il17a^{Cre}$ $Il10^{Flox/Flox}$ mice and were cultured under non-inflammatory (IL-6 + TGF-β) or inflammatory (IL-6 + IL-23 + IL-1β) T_H17 conditions. IL-10 in the supernatant was measured by ELISA on day 5. Each dot represents one replicate (*n*=3). Mean ± S.D.; ns, not significant; *P < 0.05 by Mann-Whitney U test. E. Flow cytometric analysis of *in vitro* cultured T_H17 cells. Naive $CD4^+$ T cells were isolated from the spleens of $Il17a^{Cre}$ $Il10^{Wt/Wt}$ as well as $Il17a^{Cre}$ $Il10^{Flox/Flox}$ mice and were cultured under non-inflammatory (IL-6 + TGF-β) T_H17 conditions. Cells were re-stimulated and intracellularly stained for IL-10 on Day 5. On the right the statistics of $IL\text{-}10^+$ cells within YFP^+ cells are reported. Each dot represents one separate T_H17 cell culture ($n_{wild\ type}=4$, $n_{KO}=8$) obtained from pooled naïve T cells coming from two mice. Mean ± S.D.; Mann-Whitney U-test. F. Representative histological pictures of H&E stained small intestines under steady states. One representative experiment of two is shown. Each dot represents one mouse ($n_{wild\ type}=4$, $n_{KO}=3$). Mean ± S.D.; ns, not significant by Welch's t-test. G. Frequencies of different IL-10-producing lymphocyte populations that are derived from IL-17A producers. Intestinal lymphocytes were harvested from steady state $Fate^+$ mice (Gagliani et al., *Nature 523*, 224-224, 2015). Different IL-10-producing immune cell populations that have activated $IL\text{-}17A^{CRE}$ (indicated by YFP expression) were analyzed by flow cytometry.

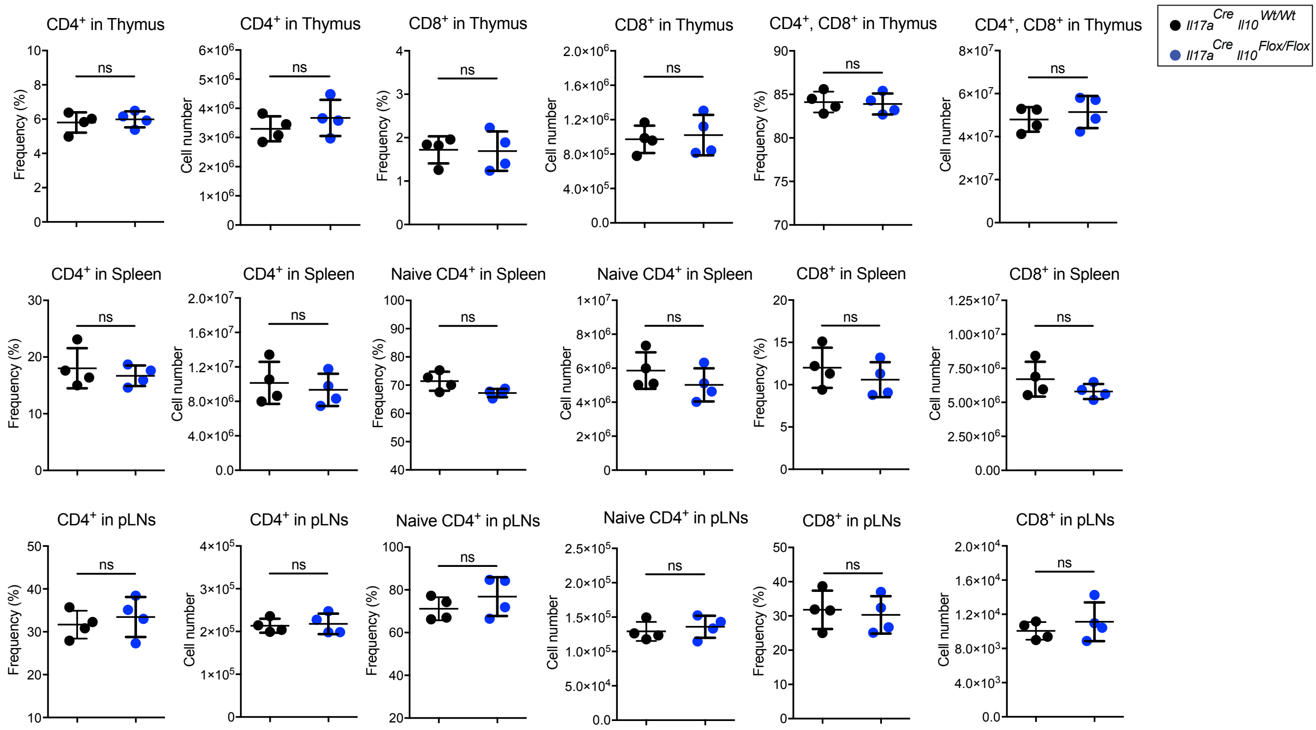

Supplementary Figure 2. No immune abnormality observed in the periphery. Frequencies and cell numbers of different T cell subsets in different immune organs. Lymphocytes from thymus, spleen and peripheral lymph nodes (pLNs) were harvested from steady state *Il17a*^{Cre} *Il10*$^{Flox/Flox}$ mice as well as their littermate wild-type control mice. Different T cell subsets were then analyzed by flow cytometry. Each dot represents one mouse ($n_{wild\ type}=4$, $n_{KO}=4$). Mean ± S.D.; ns, not significant by Mean ± S.D.; ns, not significant by Mann-Whitney *U* test.

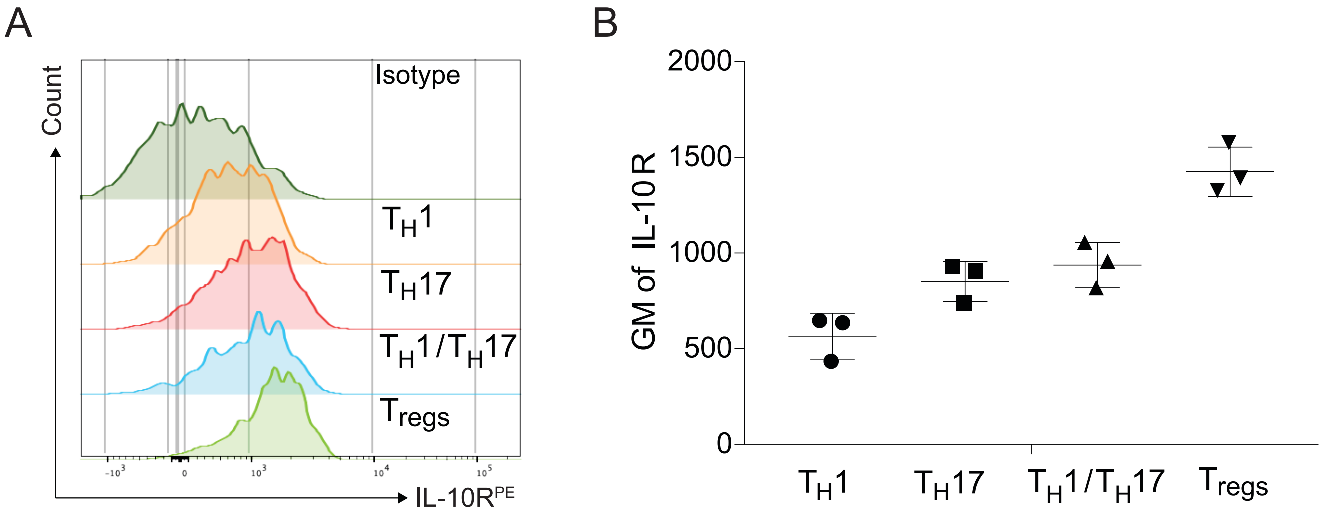

Supplementary Fig. 3 T_H17 cells express higher levels of IL-10R compared to T_H1 cells. A. Histograms showing the expression of IL-10R for T_H1 cells (defined as $CD4^+$ $CD44^+$ IFN-$\gamma^{Katushka+}$ IL-17a^{eGFP-}), T_H17 cells (defined as $CD4^+$ $CD44^+$ IFN-$\gamma^{Katushka-}$ IL-17a^{eGFP+}), T_H1/T_H17 cells (defined as $CD4^+$ $CD44^+$ IFN-$\gamma^{Katushka+}$ IL-17a^{eGFP+}) and Tregs (defined as $CD4^+$ Foxp3^{RFP+} IFN-$\gamma^{Katushka-}$ IL-17a^{eGFP-}) isolated from the intestinal lamina propria of a representative Foxp3^{RFP} IL-17a^{eGFP} IFN-$\gamma^{Katushka}$ reporter mouse injected with anti-CD3 mAb. B. Geometric Mean (GM) values of IL-10R from different CD4 T cell subsets. Each dot represents one mouse (*n=3*). Mean ± S.D.

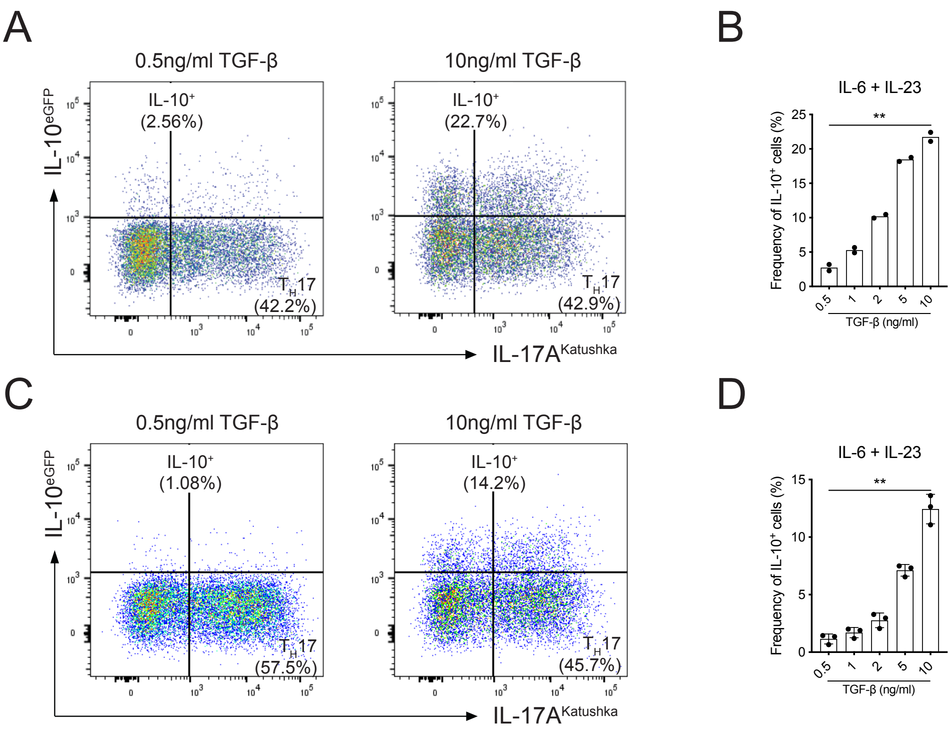

Supplementary Fig. 4 TGF-β promotes IL-10 production in mature T_H17 cells. A, C. T_H17 cells generated *in vitro* under either TGF-β condition (IL-6 + IL-23 + TGF-β) (A) or IL-1β condition (IL-6 + IL-23 + IL-1β) (C) were purified and then re-activated with IL-6, IL-23 as well as different amounts of TGF-β. Cells in the above dot plots are pre-gated on viable $CD4^+$ T cells. B, D. Statistical analysis of the frequencies of IL-10^+ cells in A, C respectively across different TGF-β conditions. **P < 0.01, by ordinary one-way ANOVA.

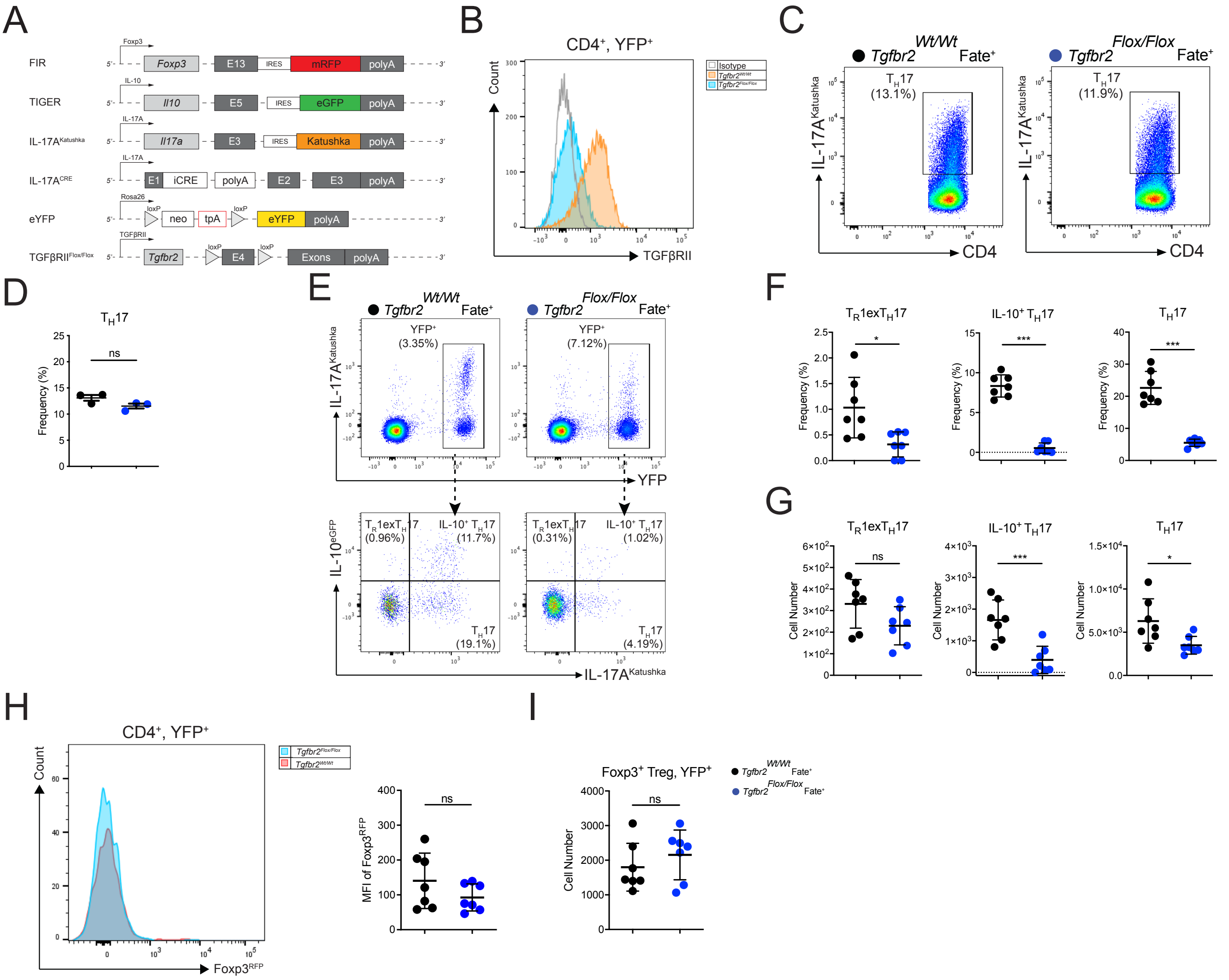

Supplementary Fig. 5 Tgfbr2 is required for T_H17 cells to produce IL-10. A. Constructs contained in Tgfbr2$^{fl/fl}$ Fate$^+$ mouse line. B. Flow cytometry analysis of TGF-βRII expression in intestinal CD4$^+$ T cells expressing YFP (indicating CRE activation). One representative of two independent experiments is shown. C. Flow cytometry analysis of naive CD4$^+$ T cells polarized under T_H17 condition *in vitro*. Cells are pre-gated on viable CD4$^+$ T cells. D. Frequency of T_H17 cells shown in C. One representative of two independent experiments is shown. Mean ± S.D.; ns, not significant by Welch's t-test. E. Flow cytometry analysis of small intestinal CD4$^+$ T cells under steady state. Top panel is pre-gated on CD4$^+$ Foxp3$^-$ T cells and bottom panel on CD4$^+$ Foxp3$^-$ YFP$^+$ T cells. The cell populations indicated on the FACS dot plots are identified by different combination of reporter expression (T_R1exT_H17: IL-10^{eGFP+} YFP$^+$ IL17a^{Kata-}; IL-10$^+$ T_H17: IL-10^{eGFP+} YFP$^+$ IL17a $^{Kata+}$; T_H17: IL-10^{eGFP-} YFP$^+$ IL17a^{Kata+}). F, G. Frequencies (F) and numbers (G) of T_R1^{exTH17}, IL-10$^+$ T_H17 and T_H17 cells. Data are pooled from three independent experiments. Each dot represents one mouse ($n_{wild\ type}$=7, n_{KO}=7). Mean ± S.D.; ns, not significant; *P < 0.05, ***P < 0.001 by Mann-Whitney U test. H. Left, Flow cytometric analysis of the Foxp3 expression level among the intestinal YFP$^+$ CD4$^+$ T cells under steady state conditions. Foxp3 expression was determined by RFP reporter fluorescence. A pre-gate on YFP$^+$ CD4$^+$ T cells is applied. Right panel shows the statistical analysis of Foxp3 Mean Fluorescence Intensity (MFI) among YFP$^+$ CD4$^+$ T cells. I. Numbers of YFP$^+$ Foxp3$^+$ Treg cells in the small intestine under steady state conditions in the two indicated mouse lines. Each dot represents one mouse ($n_{wild\ type}$=7, n_{KO}=7). Mean ± S.D.; ns, not significant by Mann-Whitney U test.

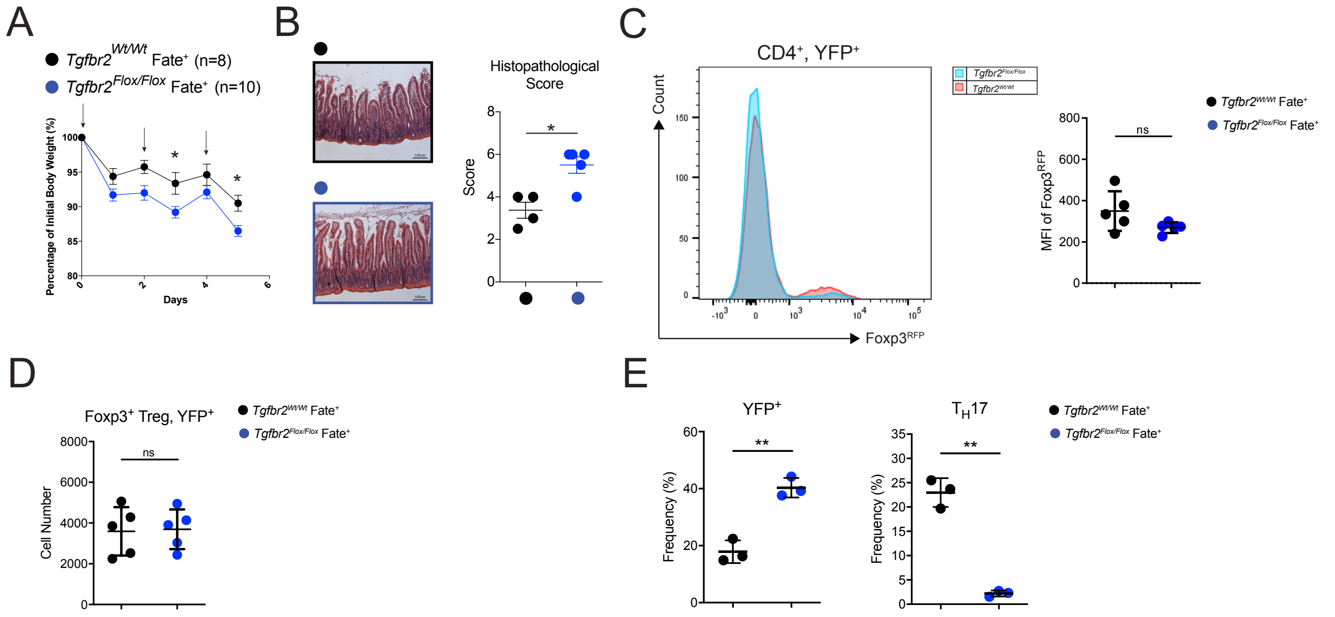

Supplementary Fig. 6 Tgfbr2 is required for T_H17 cells to produce IL-10 during intestinal inflammation. A. Percentages of initial body weight of mice after anti-CD3 mAb treatment. The arrows indicate the injection of anti-CD3 mAb. Data are cumulative of three independent experiments. Mean ± SEM.; *P < 0.05, by 2-way ANOVA with Bonferroni's post-test. B. Representative histological pictures of H&E stained small intestines after anti-CD3 treatment. Statistical analysis is representative of two experiments. Each dot represents one mouse ($n_{wild\ type}=4, n_{KO}=5$). Mean ± SEM, *P < 0.05 by Mann-Whitney U test. C. Left, flow cytometric analysis of Foxp3 expression among YFP^+ $CD4^+$ T cells in the small intestine during anti-CD3 mAb-induced intestinal inflammation. Foxp3 expression was determined by RFP reporter fluorescence. A pre-gate on YFP^+ $CD4^+$ T cells is applied. Right panel shows the statistical analysis of Foxp3 Mean Fluorescence Intensity (MFI) among YFP^+ $CD4^+$ T cells between the two indicated mouse lines. Each dot represents one mouse ($n_{wild\ type}=5, n_{KO}=5$). D. Numbers of YFP^+ $Foxp3^+$ Treg cells in the small intestine in the two indicated mouse lines. Each dot represents one mouse ($n_{wild\ type}=5, n_{KO}=5$). Mean ± S.D.; ns, not significant by Mann-Whitney U test. E. As part of Fig.4G, here it is reported the statistical analysis of YFP^+ and T_H17 cells after $CD4^+$ T cells transfer. $CD4^+$ T cells were isolated form the indicated mouse lines. Each dot represents one mouse ($n_{wild\ type}=3, n_{KO}=3$). Mean ± S.D.; **P < 0.01 by Welch's t-test.

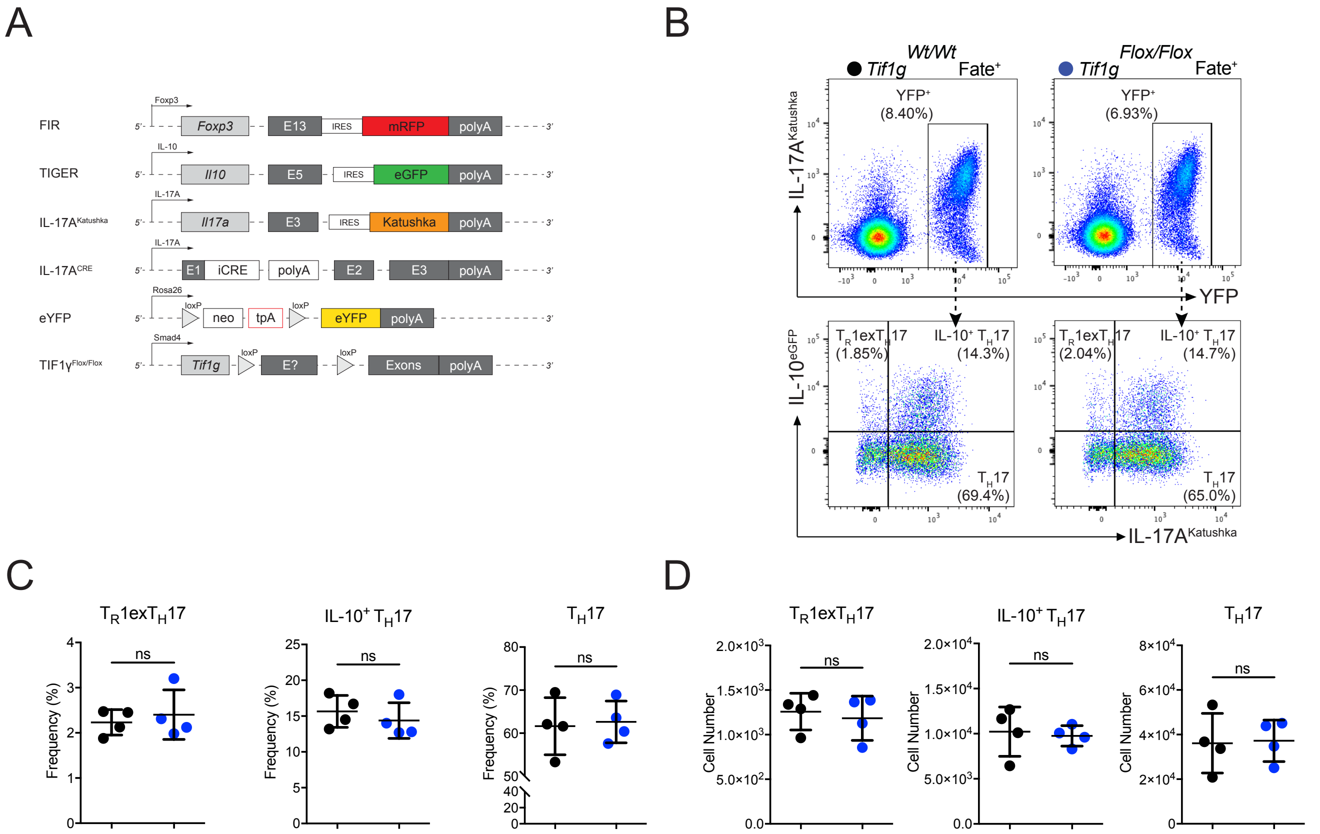

Supplementary Fig. 7 TIF1γ is dispensable for IL-10 production in T_H17 cells. A. Constructs contained in Tif1g$^{fl/fl}$ Fate$^+$ mice. B. Flow cytometry analysis of small intestinal CD4$^+$ T cells after anti-CD3-induced intestinal inflammation. Top panel, pre-gated on Foxp3$^-$ CD4$^+$ T cells; bottom panel, pre-gated on YFP$^+$ Foxp3$^-$ CD4$^+$ T cells. The populations indicated on the FACS dot plots are identified by different combination of reporter expression (T_R1exT_H17: IL-10^{eGFP+} YFP$^+$ IL17a^{Kata-}; IL-10$^+$$T_H17$: IL-10^{eGFP+} YFP$^+$ IL17a^{Kata+}; T_H17: IL-10^{eGFP-} YFP$^+$ IL17a^{Kata+}). C, D. Frequencies (C) and numbers (D) of T_R1^{exTH17}, IL-10$^+$ T_H17 and T_H17 cells. One representative of three independent experiments is shown. Each dot represents one mouse ($n_{wild\ type}=4$, $n_{KO}=4$). Mean ± S.D.; ns, not significant by Mann-Whitney U test.

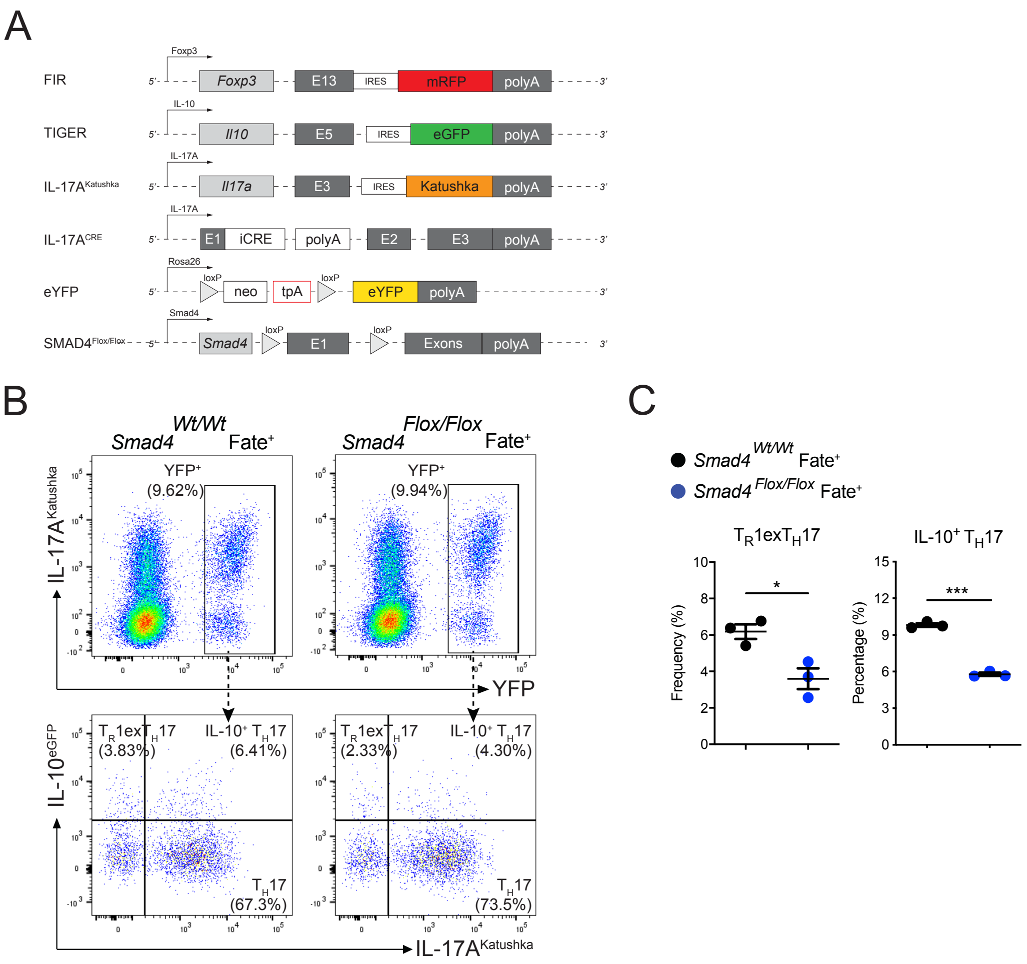

Supplementary Fig. 8 Smad4 is required for T_H17 cells to produce IL-10. A. Constructs contained in Smad4$^{fl/fl}$ Fate$^+$ mice. B. Flow cytometric analysis of *in vitro* cultured naive CD4$^+$ T cells. Cells were polarized under T_H17 condition for 5 days. Top panel, pre-gated on Foxp3$^-$ CD4$^+$ T cells; bottom panel, pre-gated on YFP$^+$ Foxp3$^-$ CD4$^+$ T cells. The populations indicated on the FACS dot plots are identified by different combination of reporter expression (T_R1exT_H17: IL-10^{eGFP+} YFP$^+$ IL17a^{Kata-}; IL-10$^+T_H17$: IL-10^{eGFP+} YFP$^+$ IL17a^{Kata+}; T_H17: IL-10^{eGFP-} YFP$^+$ IL17a^{Kata+}). B. Statistical analysis of T_R1exT_H17, IL-10$^+$ T_H17 and T_H17 cells. Data are representative of one out of three experiments. Each dot represents one mouse ($n_{wild\ type}=3$, $n_{KO}=3$). Mean ± S.D.; *P < 0.05, ***P < 0.001 by Welch's t-test.

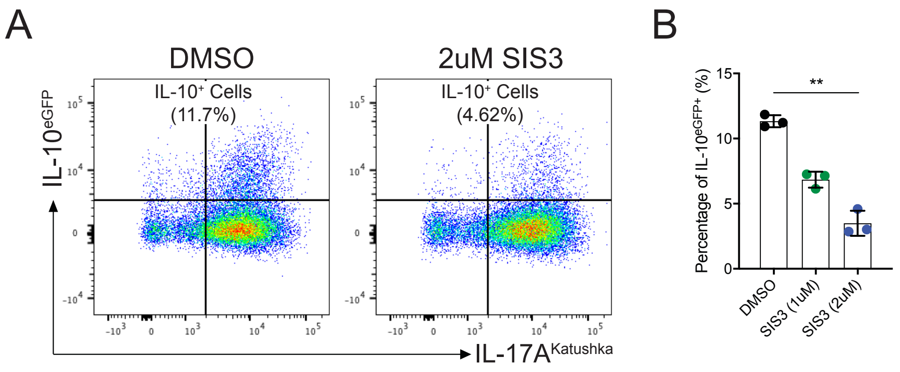

Supplementary Fig. 9 Smad3 is important for IL-10 production in T_H17 cells. *In vitro* differentiated T_H17 cells were purified and cultured in the presence 2ng/ml TGF-β together with DMSO or different amount of SIS3. A. Representative flow cytometry analysis of DMSO and 2uM SIS3 conditions. Cells were pre-gated on viable $CD4^+$ T cells. Expression of IL-10^{eGFP} was detected by reporter fluorescence. B. Statistical analysis of the frequencies of IL-10^+ cells across different conditions. Each dot represents one replicate (*n=3*). Mean ± S.D.; **P < 0.01 by Kruskal-Wallis test.

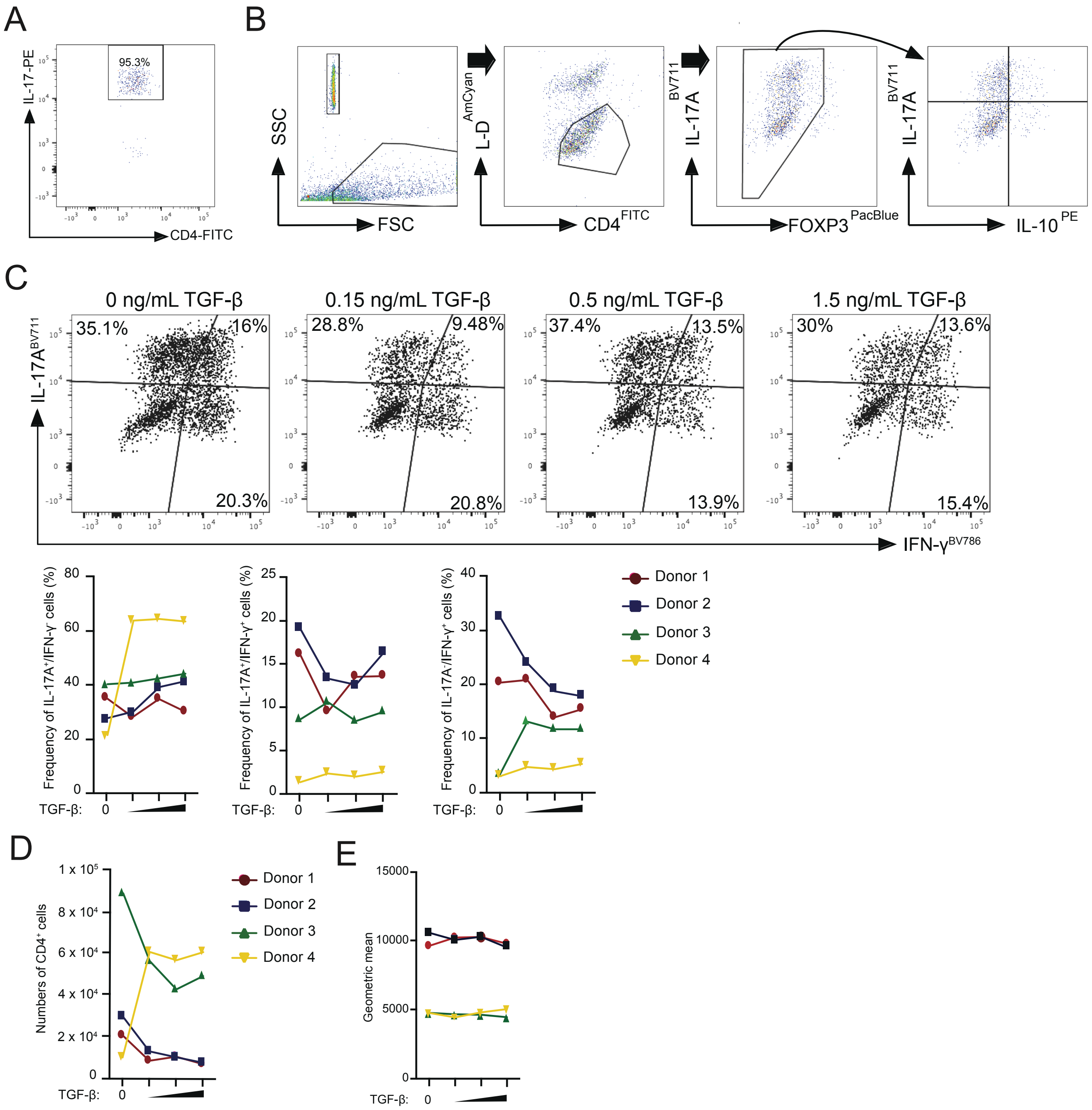

Supplementary Fig. 10 Human intestinal T_H17 cells produce IL-10 and TGF-β further promotes IL-10 expression. A. The dot plot shows the purity of the sorted $CD4^+$ $IL17A^+$ cells corresponding to blood donor 1 shown in Fig. 7. B. Gating strategy of human T_H17 cells isolated from PBMCs of one representative healthy human donor. This strategy applies to the data shown here and in Figure 7B. C. Dot plots of PBMC-sorted T_H17 cells from the human blood, stimulated and cultured under increasing TGF-β1 concentrations four 5 days and stained for IL-17A and IFN-γ. The graphs below show the percentages of IL-17A$^+$, IL-17A$^+$/IFN-γ$^+$, and IFN-γ$^+$ cells from four different blood donors. Each colored line represents one donor (*n=4*). D. Absolute total numbers of living $CD4^+$ T cells corresponding to (C). E. Foxp3 expression (MFI) among the *in vitro* cultured human T_H17 cells shown in Fig. 7, in the presence of increasing TGF-β concentrations.

Supplementary Table 1: Table of Antibodies

Name	***Clone***	***Company***	***Cat. Number***	***Use***
Anti-Smad3 (phosphoS423+S425)	EP823Y	Abcam	ab52903	WB, PLA
Anti-Smad3	-	Abcam	ab52903	ChIP
Smad4 Monoclonal Antibody	4G1C6	ThermoFischer Scientific	MA5-15682	WB, PLA, ChIP
Anti-Smad4	EP618Y	Abcam	ab40759	ChIP
Anti-acetyl Histone H4	-	Merck	06-866	WB
Pac Blue anti-mouse CD4	GK1.5	Biolegend	100428	FACS
Alexa Fluor® 700 anti-mouse CD4	RM4-5	Biolegend	100536	FACS
PE.Cy5 anti-mouse CD8	53-6.7	Biolegend	100722	FACS
PE.Cy7 anti-mouse NK1.1	PK136	Biolegend	108713	FACS
PE anti-mouse CD19	6D5	Biolegend	115508	FACS
PE.Cy7 anti-mouse CD11b	M1/70	Biolegend	101216	FACS
PE.Cy7 anti-mouse CD11c	N418	Biolegend	117318	FACS
PE.Cy7 anti-mouse γδTCR	GL3	Biolegend	118123	FACS
PE anti-mouse TGFβRII	-	R&D Systems	FAB532P	FACS
BV421 anti-mouse IL-17A	TC11-18H10.1	Biolegend	506925	FACS
PE anti-mouse IFN-γ	XMG1.2	Biolegend	554412	FACS
FITC anti-human CD4	OKT4	Biolegend	317408	FACS
Alexa Fluor® 700 anti-human CD45RA	HI100	Biolegend	304120	FACS
PE/Cy7 anti-human CD127	019D5	Biolegend	351320	FACS
Brilliant Violet 650™ anti-human CD25	BC96	Biolegend	302633	FACS
Brilliant Violet 711™ anti-human IL-17A	BL168	Biolegend	512328	FACS
PE anti-human IL-10	JES3-19F1	Biolegend	506804	FACS
Pacific Blue™anti-mouse CD4	RM4-5	Biolegend	100531	FACS

WB: Western Blot

PLA: Proximity Ligation Assay

ChIP: Chromatin Immunoprecipitation

FACS: Fluorescent Activation Cell Sorting

Supplementary Table 2: Table of primers

Name	***Sequence***	***Amplicon size***	***Use***
Il10pr Fw	5’ gttgcttctgctgttggaaacg 3’	*1447bp*	PCR, CL
Il10pr Rev	5’ gtagacctcctgttcttggtccc 3’		PCR, CL
Il10pr-pGL2 Fw	5’ gcgtcgtagctcgaggttgcttctgctgttggaaacg 3’	*1477bp*	PCR, CL
Il10pr-pGL2 Rev	5’ cggaatgccaagcttgtagacctcctgttcttggtccc 3’		PCR, CL
Smad3 Fw	5’ atgtcgtccatcctgcccttcac 3’	*1278bp*	PCR, CL
Smad3 Rev	5’ ctaagacacactggaacagcggatg 3’		PCR, CL
Smad3-pT$_N$T Fw	5’ ctttttgcactcgagatgtcgtccatcctgcccttcac 3’	*1308bp*	PCR, CL
Smad3-pT$_N$T Rev	5’ cgggtcgactctagactaagacacactggaacagcggatg 3’		PCR, CL
Smad4 Fw	5’ atggacaatatgtctataacaaatacaccaa 3’	*1656bp*	PCR, CL
Smad4 Rev	5’ tcagtctaaaggctgtgggtccg 3’		PCR, CL
Smad4-pTag2 Fw	5’ cgggctgcaggaattcatggacaatatgtctataacaaatacaccaa 3’	*1687bp*	PCR, CL
Smad4-pTag2 Rev	5’ cgtatcgataagctttcagtctaaaggctgtgggtccg 3’		PCR, CL
Il10A Fw	5’ caggttgagtggaggaaacaa 3’	*138bp*	ChIP
Il10A Rev	5’ ggcagacagctgttctatgt 3’		ChIP
Il10B Fw	5’ gcccagggtacagaatgaaa 3’	*173bp*	ChIP
Il10B Rev	5’ agctgttgaaggatggagatg 3’		ChIP
Il10C Fw	5’ agcccatttatccacgtcatta 3’	*94bp*	ChIP
Il10C Rev	5’ actggtcggaatgaacttctg 3’		ChIP
Gapdh Fw	5’ accagggagggctgcagtcc 3’	*237bp*	ChIP
Gapdh Rev	5’ tcagttcggagcccacaggc 3’		ChIP

PCR: Polymerase Chain Reaction

CL: Cloning

ChIP: Chromatin Immunoprecipitation
