# Supplementary material for: The induction and function of the anti-inflammatory fate of TH17 cells
Source: Nat Commun. 2020 Jul 3;11:3334. doi: 10.1038/s41467-020-17097-5 (PMC7335205; doi:10.1038/s41467-020-17097-5)
Supplement: Supplementary file 2 — Reporting Summary [file 41467_2020_17097_MOESM2_ESM.pdf]

nature research

Corresponding author(s): Nicola Gagliani
Last updated by author(s): May 14, 2020

# Reporting Summary

Nature Research wishes to improve the reproducibility of the work that we publish. This form provides structure for consistency and transparency in reporting. For further information on Nature Research policies, see Authors & Referees and the Editorial Policy Checklist .

## Statistics

For all statistical analyses, confirm that the following items are present in the figure legend, table legend, main text, or Methods section.

| n/a | Confirmed | |
|---|---|---|
| ☐ | ☒ | The exact sample size (*n*) for each experimental group/condition, given as a discrete number and unit of measurement |
| ☐ | ☒ | A statement on whether measurements were taken from distinct samples or whether the same sample was measured repeatedly |
| ☐ | ☒ | The statistical test(s) used AND whether they are one- or two-sided<br>*Only common tests should be described solely by name; describe more complex techniques in the Methods section.* |
| ☐ | ☒ | A description of all covariates tested |
| ☐ | ☒ | A description of any assumptions or corrections, such as tests of normality and adjustment for multiple comparisons |
| ☐ | ☒ | A full description of the statistical parameters including central tendency (e.g. means) or other basic estimates (e.g. regression coefficient) AND variation (e.g. standard deviation) or associated estimates of uncertainty (e.g. confidence intervals) |
| ☐ | ☒ | For null hypothesis testing, the test statistic (e.g. *F*, *t*, *r*) with confidence intervals, effect sizes, degrees of freedom and *P* value noted<br>*Give P values as exact values whenever suitable.* |
| ☒ | ☐ | For Bayesian analysis, information on the choice of priors and Markov chain Monte Carlo settings |
| ☐ | ☒ | For hierarchical and complex designs, identification of the appropriate level for tests and full reporting of outcomes |
| ☒ | ☐ | Estimates of effect sizes (e.g. Cohen's *d*, Pearson's *r*), indicating how they were calculated |

*Our web collection on statistics for biologists contains articles on many of the points above.*

## Software and code

Policy information about availability of computer code

| | |
|---|---|
| Data collection | BD FACS Diva (8.0.1) was used for data collection during flow cytometry analysis<br>BD Aria Fusion or BD Aria Illusion was used for Fluorescent activated cell sorting<br>BD LSR II was used for flow cytometry analysis<br>FlowStar Omega microplate reader was used to measure luciferase activity in transient transfection assays |
| Data analysis | Graph design and statistical analysis was performed with Graph pad prism Version 7.0<br>Immunohistochemistry stainned sections were scanned using a digital slide scanner and the Images were analyzed using Fiji (Image J).<br>For Flow cytometry data analysis we used FlowJo 10.5.3 |

For manuscripts utilizing custom algorithms or software that are central to the research but not yet described in published literature, software must be made available to editors/reviewers. We strongly encourage code deposition in a community repository (e.g. GitHub). See the Nature Research guidelines for submitting code & software for further information.

## Data

Policy information about availability of data

All manuscripts must include a data availability statement. This statement should provide the following information, where applicable:

- Accession codes, unique identifiers, or web links for publicly available datasets
- A list of figures that have associated raw data
- A description of any restrictions on data availability

The authors declare that the data supporting the findings of this study are available within the paper and its supplementary information file

# Field-specific reporting

Please select the one below that is the best fit for your research. If you are not sure, read the appropriate sections before making your selection.

- [x] Life sciences
- [ ] Behavioural & social sciences
- [ ] Ecological, evolutionary & environmental sciences

For a reference copy of the document with all sections, see nature.com/documents/nr-reporting-summary-flat.pdf

# Life sciences study design

All studies must disclose on these points even when the disclosure is negative.

| | |
|---|---|
| Sample size | Sample size was determined based on previous work from our group with the same models and with a power analysis using Wilcoxon-Mann-Whitney test. |
| Data exclusions | No data were excluded |
| Replication | All data shown are either representative or pooled from at least two independent experiments. All replication attempts were successful. |
| Randomization | Age- and sex-matched littermates between 8 to 24 weeks of age were used for experiments. Unless specialy noted, mice were randomly assigned to different experimental groups and each cage contained animals of all different experimental groups. Both male and female mice were used in experiments. Wild type and mice with a floxed allele were allocated to specific groupprs based on their genotype and no randomization was performed, other than both genotypes and treatments were co-housed in the same cage. |
| Blinding | Investigators were blinded to group allocation during mouse histopathological analysis. |

# Reporting for specific materials, systems and methods

We require information from authors about some types of materials, experimental systems and methods used in many studies. Here, indicate whether each material, system or method listed is relevant to your study. If you are not sure if a list item applies to your research, read the appropriate section before selecting a response.

## Materials & experimental systems

| n/a | Involved in the study | |
|---|---|---|
| [ ] | [x] | Antibodies |
| [ ] | [x] | Eukaryotic cell lines |
| [x] | [ ] | Palaeontology |
| [ ] | [x] | Animals and other organisms |
| [ ] | [x] | Human research participants |
| [x] | [ ] | Clinical data |

## Methods

| n/a | Involved in the study | |
|---|---|---|
| [x] | [ ] | ChIP-seq |
| [ ] | [x] | Flow cytometry |
| [x] | [ ] | MRI-based neuroimaging |

## Antibodies

Antibodies used

Anti-Smad3 (phosphoS423+S425) EP823Y from Abcam ab52903 (used for: WB, PLA)
Anti-Smad3 from Abcam ab52903 (used for: ChIP)
Smad4 Monoclonal Antibody 4G1C6 from ThermoFischer Sc. MA5-15682 (used for: WB, PLA, ChIP)
Anti-Smad4 EP618Y from Abcam ab40759 (used for: ChIP)
Anti-acetyl Histone H4 from Merck 06-866 (used for: WB)
FITC anti-human CD4 OKT4 from Biolegend 317408 (used for FACS)
Alexa Fluor® 700 anti-human CD45RA HI100 from Biolegend 304120 (used for FACS)
PE.Cy7 anti-human CD127 019D5 from Biolegend 351320 (used for FACS)
Brilliant Violet 650™ anti-human CD25 BC96 from Biolegend 302633 (used for FACS)
Brilliant Violet 711™ anti-human IL-17A BL168 from Biolegend 512328 (used for FACS)
PE anti-human IL-10 JES3-19F1 from Biolegend 506804 (usedd for FACS)
Pacific Blue™anti-mouse CD4 RM4-5 from Biolegend 100531 (used for FACS)
Alexa Fluor® 700 anti-mouse CD4 from Biolegend 100536 (used for FACS)
PE.Cy5 anti-mouse CD8 from Biolegend 100722 (used for FACS)
PE.Cy7 anti-mouse NK1.1 from Biolegend 108713 (used for FACS)
PE anti-mouse CD19 from Biolegend 115508 (used for FACS)
PE.Cy7 anti-mouse CD11b from Biolegend 101216 (used for FACS)
PE.Cy7 anti-mouse CD11c from Biolegend 117318 (used for FACS)

| | |
|---|---|
| | PE.Cy7 anti-mouse γδTCR from Biolegend 118123 (used for FACS)<br>PE anti-mouse TGFβRII from R&D Systems FAB532P (used for FACS)<br>BV421 anti-mouse IL-17A from Biolegend 506925 (used for FACS)<br>PE anti-mouse IFN-γ from Biolegend 554412 (used for FACS)<br>Anti-CD3 was purified in house from the hybridoma clone 2c11 (used for in vivo injections 10μg per mouse and the in vitro TH17 cell differentiation at a concentration of 10μg/mL)<br>Anti-CD28 was purified in house from the hybridoma clone PV-1(used for the in vitro TH17 cell differentiation at a concentration of 2μg/mL)<br>Anti IL-4 was purified in house from a hybridoma clone 11B11 (used for the in vitro TH17 cell differentiation at a concentration of 10μg/mL)<br>Anti IFN-γ was purified in house from a hybridoma clone XMG1.2 (used for the in vitro TH17 cell differentiation at a concentration of 10μg/mL) |
| Validation | All our antibodies were validated according to manufacturer's instructions and for FACS antibodies, unstained samples were included in every run to control for autofluorescence |

# Eukaryotic cell lines

Policy information about cell lines

| | |
|---|---|
| Cell line source(s) | HEK 293 cell line was a king gift from Dr. Kristoffer Reicken in the Center of Oncology, Department of Cell Transplantation, University Medical Center, Hamburg, Germany |
| Authentication | We did not independently authenticate the HEK 293T cell line |
| Mycoplasma contamination | The cells tested negative for Mycoplasma contamination in our in aPCR detection assay performed in house |
| Commonly misidentified lines<br>(See ICLAC register) | *Name any commonly misidentified cell lines used in the study and provide a rationale for their use.* |

# Animals and other organisms

Policy information about studies involving animals; ARRIVE guidelines recommended for reporting animal research

| | |
|---|---|
| Laboratory animals | mouse: C57BL/6 Jackson Laboratories JAX:000664<br>6- to 8-week old mice (Male and Female) were used for experiments.<br>mouse: Rag1-/- Jackson Laboratories JAX:002216<br>Age- and sex-matched littermates between 8 to 24 weeks of age were used for experiments.<br>mouse: Rosa26-Cas9 Jackson Laboratories JAX:028555<br>6- to 8-week old mice (Male and Female) were used for experiments.<br>mouse: IL-10Flox/Flox mice kindly provided by Axel Roers, Technischer Uneversitat, Dresden, Germany<br>mouse: 17ACRE mice kindly provided by Briggita Stockinger, MRC National Institute of Medical research, UK<br>Age- and sex-matched littermates between 8 to 24 weeks of age were used for experiments (For all IL-17ACre IL-10Flox/Flox experiments).<br>mouse: Fate+ mice were crossed and bred in house (Gagliani, N. et al., 2015)<br>mouse: Tgfbr2Flox/Flox, Jackson Laboratories JAX:012603<br>mouse: Smad4Flox/Flox kindly provided by Elizabeth Robertson, Oxford University, UK<br>mouse: Tif1gFlox/Flox kindly provided by Vesa Kaartinen, PhD, University of Mitchigan, MI, USA<br>Age- and sex-matched littermates between 8 to 24 weeks of age were used for experiments (For all Tgfbr2Flox/Flox Fate+, Smad4Flox/Flox Fate+ and Tif1gFlox/Flox Fate+ experiments). |
| Wild animals | This study did not contain wild animals |
| Field-collected samples | This study did not contain field collected samples |
| Ethics oversight | Animal procedures were approved by the Institutional Animal Care and Use Committee (IACUC) of Yale University, New Haven, USA. Preliminary experiments were conducted to determine sample sizes, taking available recourses and ethical use into account. |

Note that full information on the approval of the study protocol must also be provided in the manuscript.

# Human research participants

Policy information about studies involving human research participants

| | |
|---|---|
| Population characteristics | The group of participants had the following characteristics: Sex (M/F): 5/9, Age (Average +/- SD):69,5 years +/- 11 |
| Recruitment | Patients were recruited from the surgical department prior to operation due to colorectal cancer.We only recruited patients that did not underwent radiotherapy or chemotherapy to avoid potential therapy-related biases. |

Ethics oversight | The study was approved by the Ethical Physicians Committee of Hamburg "Ethik-Kommision der Aertztekammer Hamburg".

Note that full information on the approval of the study protocol must also be provided in the manuscript.

# Flow Cytometry

## Plots

Confirm that:

- [x] The axis labels state the marker and fluorochrome used (e.g. CD4-FITC).
- [x] The axis scales are clearly visible. Include numbers along axes only for bottom left plot of group (a 'group' is an analysis of identical markers).
- [x] All plots are contour plots with outliers or pseudocolor plots.
- [x] A numerical value for number of cells or percentage (with statistics) is provided.

## Methodology

Sample preparation

For flow cytometry the samples were obtained mostly from small intestine, spleen, Peyer patches (PP) and mesenteric lymph nodes (MLNs). We used DTT-containing buffers to extract lymphocytes from the small intestine that were further purified with Percol gradients. Lymphocytes from spleens, PP and MLNs were isolated via mashing the organs through 40µm filters. The spleen cells were teated additionally with RBC lysis to remove the erythrocyte content. All lymphocytes were then stained according to antibody manufacturer's instructions, in dilutions determined by previous titrations and according to standard protocols.

Instrument

Flow cytometry data were collected using BD LSRFortessa instruments

Software

BD FACS Diva was used for data collection and for Flow cytometry data analysis we used FlowJo 10.5.3

Cell population abundance

The abundance of IL17A-BV711 cells in the blood of healthy human donors among

Gating strategy

For mouse samples, among single cells:
CD4 positive cells were: eFluor-506 (AmCyan) viability Dye negative and CD4-PacBlue positive cells
non FoxP3 cells were: both IL17A-katushka positive and negative cells
Fate positive cells were: IL17A-katushka positive and negative cells and YFP positive cells
YFP positive cells were subsequently allocated as
IL17A-katushka positive and IL-10 negative (TH17 cells),
IL17A-katushka positive and IL-10-GFP positive (IL-10+ TH17 cells),
IL17A-katushka negative and IL-10-GFP positive (TR1exTH17 cells),
IL17A-katushka negative and IL-10-GFP negative (exTH17 cells).
For human samples, among single cells:
CD4 positive cells were: eFluor-506 viability Dye negative and CD4-FITCH positive cells
non FoxP3 cells were: FoxP3-PacBlue negative and IL17A-BV711 positive and negative cells
non FoxP3 cells were allocated as:
IL17A-BV711 positive,
IL17A-BV711 positive and IL-10-PE positive or
IL17A-BV711 positive and IFNγ-BV786 positive and as
IL17A-BV711 negative and IL-10-PE positive cells

- [x] Tick this box to confirm that a figure exemplifying the gating strategy is provided in the Supplementary Information.
